# Creation of Al‐Enriched Mesoporous ZSM‐5 Nanoboxes with High Catalytic Activity: Converting Tetrahedral Extra‐Framework Al into Framework Sites by Post Treatment

**DOI:** 10.1002/anie.202002416

**Published:** 2020-03-26

**Authors:** Yilai Jiao, Luke Forster, Shaojun Xu, Huanhao Chen, Jingfeng Han, Xuqing Liu, Yangtao Zhou, Jinmin Liu, Jinsong Zhang, Jihong Yu, Carmine D'Agostino, Xiaolei Fan

**Affiliations:** ^1^ Shenyang National Laboratory for Materials Science Institute of Metal Research Chinese Academy of Sciences 72 Wenhua Road Shenyang 110016 China; ^2^ Department of Chemical Engineering and Analytical Science School of Engineering The University of Manchester Oxford Road Manchester M13 9PL UK; ^3^ National Engineering Laboratory for Methanol to Olefins Dalian National Laboratory for Clean Energy Dalian Institute of Chemical Physics Chinese Academy of Sciences Dalian 116023 China; ^4^ Department of Materials School of Natural Sciences The University of Manchester Oxford Road Manchester M13 9PL UK; ^5^ State Key Laboratory of Inorganic Synthesis and Preparative Chemistry College of Chemistry Jilin University Changchun 130012 China; ^6^ International Center of Future Science Jilin University 2699 Qianjin Street Changchun 130012 China

**Keywords:** ZSM-5, post-synthetic treatment, mesoporous nanoboxes, diffusion, PFG-NMR

## Abstract

ZSM‐5 zeolite nanoboxes with accessible *meso*‐micro‐pore architecture and strong acid sites are important in relevant heterogeneous catalysis suffering from mass transfer limitations and weak acidities. Rational design of parent zeolites with concentrated and non‐protective coordination of Al species can facilitate post‐synthetic treatment to produce mesoporous ZSM‐5 nanoboxes. In this work, a simple and effective method was developed to convert parent MFI zeolites with tetrahedral extra‐framework Al into Al‐enriched mesoporous ZSM‐5 nanoboxes with low silicon‐to‐aluminium ratios of ≈16. The parent MFI zeolite was prepared by rapid ageing of the zeolite sol gel synthesis mixture. The accessibility to the *meso*‐micro‐porous intra‐crystalline network was probed systematically by comparative pulsed field gradient nuclear magnetic resonance diffusion measurements, which, together with the strong acidity of nanoboxes, provided superb catalytic activity and longevity in hydrocarbon cracking for propylene production.

## Graphical abstract

**ZSM‐5 nanoboxes with low SARs**: Rapid ageing of a sol gel synthesis mixture produces a parent zeolite with tetrahedral extra framework Al and a low silicon‐to‐aluminium ratio (SAR) value of ≈12, which is transformed to mesoporous ZSM‐5 nanoboxes with low SARs of ≈16 during post‐synthetic TPAOH treatment. The mesoporous ZSM‐5 nanoboxes show comparatively enhanced mass transfer ability, catalytic cracking activity and longevity.

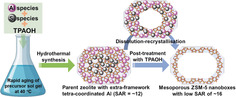

## Introduction

Hierarchical zeolites with mesoporous features are a class of inorganic materials for which previous research has confirmed the protective role of the tetrahedral framework Al for Si extraction in alkaline media. For parent ZSM‐5 zeolites with low silicon‐to‐aluminium ratios (SAR) of <20 (i.e., Al‐rich), post‐synthetic alkaline treatments are not effective for the formation of mesoporous features, and sequential fluorination‐desilication and steaming‐desilication for creating mesoporous structures in the ZSM‐5 zeolites are necessary.^1^ However, the use of corrosive chemicals and steam makes the additional treatment processes dangerous and energy‐intensive, and thus industrially unfavorable. Conversely, for highly siliceous ZSM‐5 with SAR values >50, excessive desilication occurs during post‐synthetic alkaline treatments, leading to the uncontrollable formation of mesopores.^2^ Accordingly, the optimal framework SAR of 25–50 was identified for forming mesoporous structures effectively and controllably.^1^, ^2^, ^3^ Such materials are of high industrial relevance, particularly for heterogeneous catalysis for both conventional petrochemical and the emerging biomass conversions, due to the improved diffusion properties of reactants and/or products within their crystalline domains.^4^ MFI‐type zeolites (with the nearly circular cross section of ≈0.54 nm diameter), especially ZSM‐5, are one of the most important class of active components (along with FAU‐type Y zeolites) in petroleum refining and petrochemical catalysis. They have seen significant growth in recent years for developing propylene‐selective processes such as catalytic cracking and the methanol‐to‐olefins (MTO) process.^5^ The limited accessibility of guest molecules to intrinsic micropores of ZSM‐5 is still very problematic for unfolding its full catalytic potential. ZSM‐5 normally has medium to high silicon‐to‐aluminium molar ratios (SAR ≥≈12) with heterogeneous Al distribution across its framework.3a, 6 Post‐synthetic alkaline treatment (via selective desilication) using sodium hydroxide (NaOH)^2^, 3a and sodium carbonate (Na_2_CO_3_)^7^ are generally effective to create mesoporous structures in ZSM‐5 such as mesopores and hollow crystals. Notice that SAR values of parent ZSM‐5 zeolites are the critical parameter in the controlled dissolution of Si species from the framework to form accessible *meso*‐micro‐porosity.

The use of structural directing agents (SDAs), especially tetrapropylammonium hydroxide (TPAOH), in the modification of MFI zeolites (such as TS‐1,^8^ silicalite‐1^9^ and ZSM‐5^10^) under alkaline conditions can facilitate the recrystallization of leached species from dissolution (i.e., the dissolution‐recrystallization mechanism^8^), and hence enable the controlled formation of hollow yet mesoporous zeolite crystals (i.e. nanoboxes). Such zeolite nanoboxes are important for catalysis, not only being able to allow percolation diffusion but also providing the opportunity for functionalization (e.g., encapsulation of metal nanoparticles).^10^ Since recrystallization on the outer surface of parent zeolite crystals occurs only with the pre‐selective dissolution of unprotected Si species, the dissolution‐recrystallization route is again limited by the precise location of Al in the framework of the parent zeolite; this is critical to the success of post‐synthetic desilication methods aiming to improve molecular transport through the zeolite crystals. Currently, to enable the post‐synthetic modification using SDAs for making mesoporous ZSM‐5 nanoboxes, the use of parent zeolites such as siliceous silicalite‐1 (in presence of Al sources)^9^ or ZSM‐5 with SAR values >405d, 9c, 10, 11 (ideally 40–7410b) is the essential prerequisite. Therefore, although ZSM‐5 nanoboxes with a low framework SAR of <25 (and hence high Brønsted acidity) are significantly beneficial to zeolite catalysis (especially diffusion‐limited reactions), one‐step post‐synthetic alkaline treatments of the homologous parent zeolites to obtain such materials is challenging, regardless with or without SDAs or additional Al sources. Therefore, rational design of the parent zeolite with high concentration yet non‐protective coordination of Al species (which facilitates dissolution of Si and recrystallization of Si and Al to crystallographic T‐sites during the post‐synthetic treatment) can be the solution to develop novel mesoporous ZSM‐5 with the combination of *meso*‐micro‐pore architecture and concentrated Brønsted acidity.

**Figure 1:**
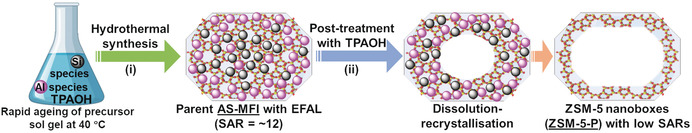
Preparation of the mesoporous ZSM‐5‐P nanoboxes via rapid ageing (of the precursor sol gel mixture) and post‐synthetic TPAOH treatment.

Herein, we report a simple yet effective method to synthesize mesoporous ZSM‐5 nanoboxes with the low SAR value of ≈16. The method involves (i) the synthesis of a parent zeolite with tetrahedral extra‐framework Al (EFAL) and (ii) post‐synthetic treatment of the parent zeolite with TPAOH solution (Figure 1). The novel feature of the method lies in the rapid ageing of the zeolite sol gel synthesis mixture at 40 °C (i.e., 30 min), which enables the synthesis of the parent zeolite with tetrahedral EFAL. The subsequent treatment using TPAOH solution allows the recrystallization and redistribution of dissolved Si and EFAL to occur, forming highly crystalline hollow ZSM‐5 zeolites with Al‐rich shell (SAR=ca. 16), affording improved acidity for catalysis. The mechanism of the new method is elaborated based on the comprehensive characterization of materials using X‐ray powder diffraction (XRD), nitrogen (N_2_) physisorption, solid‐state nuclear magnetic resonance (NMR) spectroscopy and ammonia temperature programmed desorption (NH_3_‐TPD) analysis at different stages of the synthesis. Notably, the obtained ZSM‐5 nanoboxes possess significantly high Al concentration (SAR of ≈16) in its shell (≈20 nm) and mesoporous features (e.g., specific mesopore volume≈0.26 cm^3^ g^−1^). The percolation diffusion of probing molecules within the materials was assessed by pulsed‐field gradient NMR (PFG‐NMR) measurements (Supporting Information, Figure S1). Previous research has shown that PFG‐NMR is a powerful tool of investigating the mass transport in zeolites with mesoporous features^12^ and cracking catalysts,^13^ such as intracrystalline diffusivity. However, the interpretation of the diffusion data in relation to the relevant catalytic data has not yet been reported. Benefits of the mesoporous ZSM‐5 nanoboxes to catalytic cracking reactions (of *n*‐octane and cumene) is demonstrated, showing excellent activity and selectivity to propylene due to the unique combination of pore structural features and chemical properties (i.e., the low SRA and percolating porous network). The simple and effective strategy solves the challenge of preparing mesoporous ZSM‐5 zeolites with low SAR values.

## Results and Discussion

**Figure 2:**
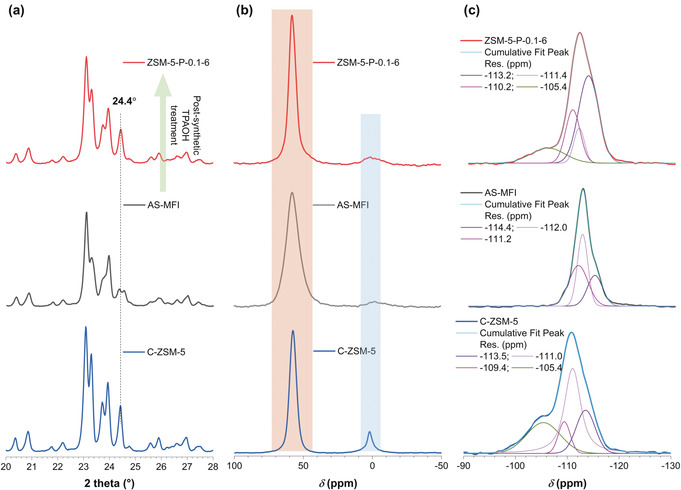
a) XRD patterns, b) ^27^Al MAS NMR spectra and c) ^29^Si MAS NMR spectra of ZSM‐5‐P‐0.1‐6 (top), AS‐MFI (middle) and C‐ZSM‐5 zeolites (bottom).

For the first time, the rapid ageing of the sol gel synthesis mixture (at 40 °C for 30 min, thereby increasing the rate of hydrolysis of the silica source tetraethylorthosilicate, TEOS) was explored to produce the as‐synthesized parent MFI zeolite (AS‐MFI) with tetrahedral EFAL (see Supporting Information for details). Comparison of X‐ray powder diffraction (XRD) patterns of AS‐MFI and conventional ZSM‐5 (C‐ZSM‐5) is shown in Figure 2 a, showing the distinct difference at 2*θ* of ≈24.4°. AS‐MFI shows the characteristics of a monoclinic phase for MFI zeolites,^14^ that is, split twin peaks, proving the presence of tetrahedral EFAL. Conversely, C‐ZSM‐5 (prepared via conventional ageing at ice bath temperature for 24 h) shows a single peak at ca. 24.4° which is assigned to the typical orthorhombic crystal symmetry of ZSM‐5.^15^ Comparably, after TPAOH treatment of AS‐MFI (pH≈13, at 160 °C under static conditions), the resulting zeolites (denoted as ZSM‐5‐P‐x‐y, where P represents via post‐treatment, *x* represents the TPAOH concentration in M and *y* represents post‐treatment time in h) show the single peak at about 24.4° as well (Figures 2 a and S2a), suggesting the re‐insertion of the tetrahedral EFAL in AS‐MFI into the newly formed framework (due to dissolution and recrystallization mechanism)^8^ during the post‐treatment. We found that a 6 h post‐treatment of AS‐MFI with 0.1 m TPAOH was sufficient to produce ZSM‐5 nanoboxes with excellent crystallinity (relative crystallinity (RC) >98 %, Figure S2b) and mesoporous features (Figure S3 and Table S1). By extending the treatment time beyond 12 h, excessive dissolution occurred, damaging the intactness of the hollow structure to certain extents (as evidenced by transmission electron microscopy (TEM) and scanning electron microscopy (SEM) analysis, Figures S4 and S5) and variation in mesoporosity of ZSM‐5‐P zeolites (Table S1). The excessive dissolution due to the prolonged treatment time (>12 h) was also reflected by the reduced RC values of the relevant ZSM‐5‐P zeolites (at ≈81 %, Figure S2b).

Solid‐state ^27^Al magic angle spinning (MAS) NMR analysis (Figure 2 b) shows the presence of tetrahedral (chemical shift centers at ≈58 ppm) and octahedral Al species (chemical shifts at about 0 ppm) for all zeolites. However, one‐dimensional ^27^Al MAS NMR is not able to elucidate the local symmetry and coordination state of Al species in zeolites.^16^ Previous research has confirmed that the change of the peak width at ≈58 ppm indicates a variation of the local Al coordination after post‐treatments of MFI zeolites.9b, 9c, 17 In this work, it was measured that the peak width of tetrahedral ^27^Al in AS‐MFI is the broadest among the samples under investigation (shaded orange area in Figure 2 b), suggesting the presence of EFAL in AS‐MFI. The post‐treatment using TPAOH changed the local Al coordination substantially, and the peak width of the resulting ZSM‐5‐P‐0.1‐6 narrowed noticeably, being comparable to that of the highly crystallized C‐ZSM‐5. Such a variation of the NMR signal of tetrahedral Al may be attributed to the reintegration of EFAL in the recrystallized framework.9b, 9c, 17 A comparison of ^29^Si MAS NMR spectra of AS‐MFI, ZSM‐5‐P‐0.1‐6 and C‐ZSM‐5 is shown in Figure 4 c. Only resonances above −110 ppm were measured for AS‐MFI, which are assigned to Si(4Si) sites,^15^ proving the insignificant presence of the framework Al in AS‐MFI. For C‐ZSM‐5, the chemical shifts of lower intensities at −103–108 ppm (corresponding to Si(1Al)) and <‐100 ppm (corresponding to Si(2Al))^18^ were identified. After the TPAOH treatment, the band at −105.4 ppm was measured for ZSM‐5‐P‐0.1‐6, evidencing the conversion of EFAL into framework T‐sites.

Post‐treatments using SDAs, especially TPAOH, are known to be effective to recover the dissolved species to a certain extent (reducing the loss of materials) and to form hollow MFI zeolites with controlled properties such as wall thickness.5d, 6, 8, 9, 10 However, for ZSM‐5, the SAR value of the parent zeolite is the most important parameter to ensure the successful dissolution‐recrystallization process. Accordingly, the formation of ZSM‐5 with regular hollow structures is only likely with the parent ZSM‐5 having the SAR value >40,5d, 10, 11 leading to hollow materials with the Si‐rich shell. According to energy dispersive X‐ray spectrometry (EDX) and inductively coupled plasma atomic emission spectrometry (ICP‐AES), the parent AS‐MFI has a bulk SAR of ≈12 (Table S1). A conventional ZSM‐5 with a low SAR of about 12 is not suitable as parent zeolite for preparing ZSM‐5 with mesoporous hollow structures via the controlled dissolution and recrystallization approach. As discussed above, AS‐MFI inherently possesses tetrahedral EFAL which does not interfere with the Si dissolution during the post‐treatment. More importantly, the co‐recrystallization of dissolved Si and EFAL facilitated by TPAOH (0.1 m at 160 °C) on the external surface during the post‐treatment (6 h to 96 h) produced zeolite nanoboxes with Al‐rich walls (SARs of ≈16, Table S1). X‐ray photoelectron spectroscopy (XPS) also shows that the surface SARs of ZSM‐5‐P zeolites are lower than the respective bulk ones detected by EDX and ICP (Table S1), suggesting the occurrence of Al redistribution during the TPAOH treatment.

**Figure 3:**
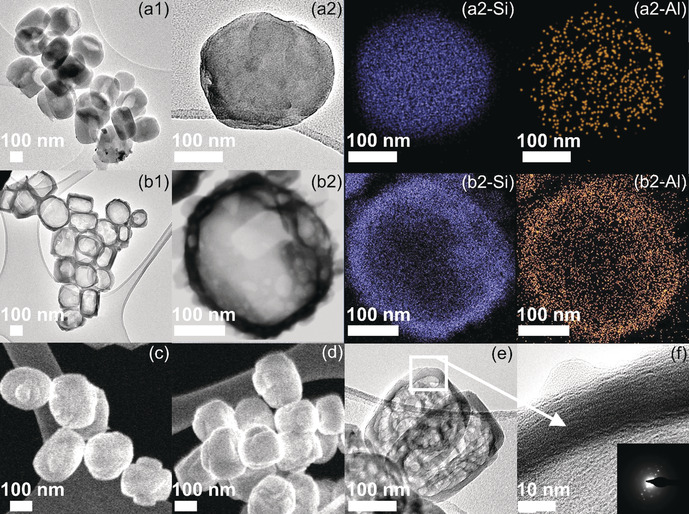
HRTEM micrographs of (a1,a2) AS‐MFI and (b1,b2) ZSM‐5‐P‐0.1‐6 zeolites; STEM micrographs of (c) AS‐MFI and (d) ZSM‐5‐P‐0.1‐6 zeolites; (e,f) HRTEM micrograph of ZSM‐5‐P‐0.1‐6 zeolite (Inset: the corresponding fast Fourier transform (FFT) of HRTEM).

**Figure 4:**
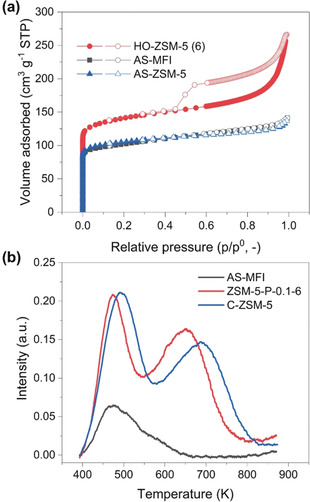
a) N_2_ adsorption (solid symbols)/desorption (open symbols) isotherms and b) NH_3_‐TPD curves of AS‐MF, C‐ZSM‐5 and ZSM‐5‐P‐0.1‐6.

AS‐MFI is primarily microporous (specific external surface area, *S*_BET_=375 m^2^ g^−1^) with uniform sizes of about 300–500 nm (as shown by the High resolution TEM (HRTEM) and scanning transmission electron microscopy (STEM) analysis, Figures 3 a1, b1 and c). After the post‐treatment, zeolite nanoboxes were formed with comparable crystal sizes (Figures 3 a1, b1, c, d and S4). The post‐treatment conditions used (i.e. 0.1 m TPAOH, at 160 °C for <12 h) were suitable to produce the regular ZSM‐5 nanoboxes with uniform cavities, which can be attributed to the preferential desilication of the siliceous part of AS‐MFI and recrystallization of dissolved Si and EFAL. The shell thickness of ZSM‐5‐P zeolites is about 20 nm (Figures 3 e,f and S4). The shell of ZSM‐5‐P‐0.1‐6 is well crystalline, as evidenced by the fast Fourier‐transform (FFT) of HRTEM (inset in Figure 3 f) and XRD analysis (Figure S2). The Al‐rich walls of ZSM‐5 nanoboxes are also confirmed by EDX, as shown in Figure 3 b2‐Al. ZSM‐5‐P nanoboxes show significant mesoporous features as revealed by nitrogen (N_2_) adsorption‐desorption analysis (Figures 4 a and S3, Table S1) with specific external surface areas (*S*_ext._)=113–149 m^2^ g^−1^ and mesopores volumes (*V*_meso._)=0.16–0.26 cm^3^ g^−1^. Using concentrated SDA (i.e. 0.3 m or 0.5 m TPAOH, Supporting Information) in the post‐treatment (24 h) was not beneficial to the formation of mesoporous hollow structures (e.g., *S*_ext._<86 m^2^ g^−1^), as well as reducing the crystallinity of the resulting zeolites (i.e., ZSM‐5‐P‐0.3‐24 and ZSM‐5‐P‐0.5‐24, RC <56 %), which is evidenced by various characterization data of the materials (Figures S6–S9 and Table S2). This is again due to the fast and excessive dissolution, which suppresses the recrystallization rate, making the formation of mesoporous hollow structures challenging. The RC value of ZSM‐5‐P‐0.5‐24 from the treatment using 0.5 m TPAOH aqueous solution was only ≈51 % (Table S2), suggesting significant loss of crystallinity due to the fast dissolution. N_2_ physisorption analysis also shows that the hysteresis loops of ZSM‐5‐P‐0.3‐24 and ZSM‐5‐P‐0.5‐24 zeolites are less significant (Figure S8) in comparison to that of ZSM‐5‐P zeolites (Figure S3). In summary, post‐treatment with TPAOH solution is effective to revive EFAL in the parent AS‐MFI, converting it into framework Al in ZSM‐5‐P zeolites. However, the balance of dissolution and recrystallization needs to be regulated (by varying the treatment time and the concentration of aqueous TPAOH solution) in order to obtain well‐defined crystalline nanoboxes. C‐ZSM‐5 shows typical features of conventional ZSM‐5 zeolites which were characterized as presented in Table S2. As discussed above, the post‐synthetic treatment of C‐ZSM‐5 (with 0.1 m TPAOH for 6 h, Supporting Information) did not result in the development of mesoporous structures (e.g. *S*_ext._≈76 m^2^ g^−1^, Figures S10–S12, Table S2) due to the abundant presence of framework Al as shown by solid state NMR (Figure S13), inhibiting the effective dissolution of Si, and resulting in the post‐treated ZSM‐5 zeolite (i.e., P‐C‐ZSM‐5‐0.1 (6)) with limited and irregular mesopores as shown by SEM and TEM (Figure S11).

Al‐rich ZSM‐5‐P nanoboxes (i.e., with low SARs) show improved acidity, especially strong acidity corresponding to Brønsted acidity (concentration of strong acid sites, as determined by NH_3_‐TPD for NH_3_ desorption at 300–500 °C, Figure S14 and Table S3), which is beneficial to catalysis. Due to the absence of Al−O−Si sites in AS‐MFI, its strong acidity is insignificant at 24.1 mmol g^−1^, as shown in Figure 4 b and Table S3 and infrared (IR) study of pyridine adsorption on zeolites (Figure S15). It should be noted that the lack of strong acidity in AS‐MFI also serves as evidence for the absence of framework tetrahedral Al in it. Conversely, ZSM‐5‐P nanoboxes show a significantly enhanced acidity (with an average strong acidity of 404.9±33.4 mmol g^−1^), reflecting the reconstruction of EFAL to tetra‐coordinated framework Al during the post‐treatment. Regarding the acidity of C‐ZSM‐5, it is comparable to that of ZSM‐5‐P nanoboxes (Figure S14c and Table S3). However, the post‐treatment of C‐ZSM‐5 (to P‐C‐ZSM‐5‐0.1 (6)) reduced the strong acidity by ca. 28 % (from 393.6 to 282.3 mmol g^−1^, Table S3).

ZSM‐5‐P nanoboxes combining the mesoporous structure with the low SAR value (≈16, corresponding to a high concentration of framework Al) favor relevant zeolite catalyzed reactions such as propylene‐selective catalytic cracking. ZSM‐5 is a widely used additive in cracking catalysis for improving propylene selectivity,5b due to its pore diameter providing size/shape selectivity. However, the conventional microporous ZSM‐5 zeolite is prone to deactivation due to carbon deposition which is mainly the results of long diffusion pathways, as well as redundant strong acid sites.^19^ Although strong acid sites are crucial for catalytic reactions, especially for hydrocarbon cracking, the reactants could over‐react on the acid sites and convert to aromatic hydrocarbons or deposit on the zeolite, resulting in the deactivation of the catalyst. Therefore, the development of zeolite catalysts combing strong acidity with improved accessibility to active sites will be highly beneficial to address this challenge.

**Figure 5:**
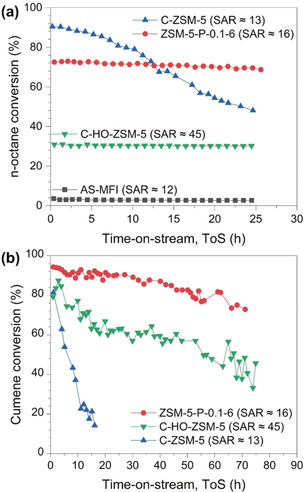
Conversion profiles of different zeolites as a function of time‐on‐stream (ToS) in a) catalytic *n*‐octane cracking and b) catalytic cumene cracking.

Comparative catalytic evaluation of ZSM‐5‐P‐0.1‐6 along with the control catalysts AS‐MFI, C‐ZSM‐5, and a conventional hollow ZSM‐5 nanoboxes with a SAR value of ≈45 (i.e. C‐HO‐ZSM‐5, Figures S16 and S17, Table S4, Supporting Information) was performed using cracking reactions with *n*‐octane (kinetic diameter (KD)=0.43 nm)^20^ and cumene (KD=0.68 nm)^21^ as model naphtha and aromatic compounds (Figures 5 and S18, Tables S5 and S6). Catalysis was carried out in a fixed bed reactor (I.D.=10 mm) with 1 g pelletized zeolite catalysts (particle size=1.6–1.8 mm). Figure 5 presents the conversion of *n*‐octane over different zeolites at 540 °C as a function of time‐on‐stream (ToS). AS‐MFI shows insignificant activity compared to other catalysts due to the lack of framework Al, and thus Brønsted acidity (Figures S14a and S15). Although the microporous C‐ZSM‐5 presented the highest initial activity (with an initial *n*‐octane conversion of ≈90 %), it deactivated gradually and significantly over time (the final *n*‐octane conversion and dropped to ≈48 % after 25 h on stream). The deactivation of C‐ZSM‐5 was due to coke formation on the external surface of the crystals, which was the result of the diffusion resistance caused by the pure microporous framework of C‐ZSM‐5, leading to the loss of accessibility and acidity (i.e., *S*_BET_ and strong acidity dropped by about 73 % and 57 %, respectively, according to the post‐reaction characterization of the used catalysts using N_2_ physisorption and NH_3_‐TPD analyses, Tables S7 and S8). Conversely, the ZSM‐5‐P‐0.1‐6 nanoboxes promoted the diffusion of *n*‐octane through their newly formed percolation pore network, being highly stable regarding both *n*‐octane conversion (at ca. 73 %) and selectivity to propylene (at ≈30 %, as shown in Figure S18). C‐HO‐ZSM‐5 with the mesoporous hollow structure (Figures S16 and S17) showed a stable catalytic performance in cracking *n*‐octane as well. However, due to the low concentration of strong acidity in C‐HO‐ZSM‐5 (at 214.4 mmol g^−1^), it was outperformed by ZSM‐5‐P‐0.1‐6 nanoboxes by ≈130 % regarding *n*‐octane conversion. The used ZSM‐5‐P‐0.1‐6 (denoted as ZSM‐5‐P‐0.1‐6‐U) can be regenerated by calcination at 550 °C under 10 vol. % O_2_ in N_2_. The regenerated ZSM‐5‐P‐0.1‐6 showed comparable chemical, physical and catalytic properties, as shown in Figures S19–S21 and Tables S7,S8).

The accessibility issue was substantial when relatively bulky cumene (KD=0.68 nm)^21^ was cracked (at 320 °C). The advantages of Al‐rich ZSM‐5‐P‐0.1‐6 nanoboxes over the conventional microporous C‐ZSM‐5 and conventional C‐HO‐ZSM‐5 (SAR≈45) are highly recognizable. Severe deactivation (i.e., cumene conversion dropped by ≈88 % within 10 h) was measured for C‐ZSM‐5 due to coke deposition (as shown by thermogravimetry, N_2_ physisorption and NH_3_‐TPD analysis of the used zeolite catalysts, Figure S22, Tables S9,S10). By comparing the two ZSM‐5 nanoboxes under study, ZSM‐5‐P‐0.1‐6 showed remarkably better activity than C‐HO‐ZSM‐5 on stream of 70 h (e.g., deactivation rate regarding the cumene conversion: −0.31 % h^−1^ for ZSM‐5‐P nanoboxes vs. −0.48 % h^−1^ for C‐HO‐ZSM‐5). More importantly, although with a low SAR value of ≈16, ZSM‐5 nanoboxes remained stable as well, as evidenced by the comparable XRD and N_2_ physisorption analysis of the used ZSM‐5‐P‐0.1‐6 before and after steam ageing (at 500 °C for 10 h with 50 % water in N_2_, Figures S23 and Table S11). The specific micropore surface area (*S*_micro_) of the used ZSM‐5‐P‐0.1‐6 after steam ageing dropped by ca. 6 % (from 415 m^2^ g^−1^ to 391 m^2^ g^−1^), while the RC values remained comparable at ≈85 %.

**Figure 6:**
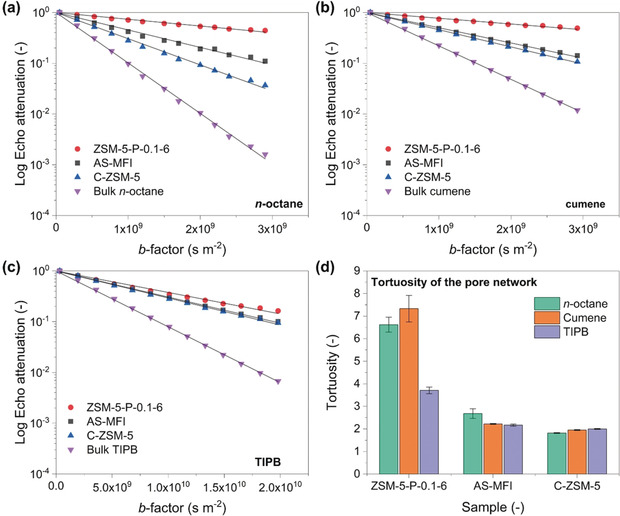
PFG‐NMR log attenuation plots for a) *n*‐octane, b) cumene and c) TIPB within different zeolite samples under investigation [solid lines represent the fittings using Eq. (S1)]; d) Values of tortuosity of the probe molecules of *n*‐octane, cumene and TIPB in different zeolite samples.

As previously mentioned, it is likely that the creation of an intra‐crystalline accessible *meso*‐micro‐porous structure of ZSM‐5‐P nanoboxes contributes to the measured catalytic activity; this was experimentally confirmed by PFG‐NMR measurements carried out at a ^1^H frequency of 43 MHz, with a diffusion probe capable of producing magnetic field gradient pulses up to 163 mT m^−1^, at atmospheric pressure and 25 °C. The mass transport properties of the zeolites under study measured by PFG‐NMR (using probing molecules of *n*‐octane, cumene and 1,3,5‐triisopropylbenzene, TIPB) are shown in Figure 6, together with PFG‐NMR plots of the bulk liquid of probing molecules (i.e., purple inverted triangle symbols in Figures 6 a–c) as a reference. Diffusion measurements were performed using the pulsed‐field gradient stimulated echo sequence (PGSTE sequence).^22^ The sequence is made by combining a series of radiofrequency pulses (RF) with magnetic field gradients (*g*, Figure S1). According to Eq. (S1), the NMR signal attenuation of PFG‐NMR experiments as a function of the gradient strength, *E*(*g*), is related to the experimental variables and the diffusion coefficient [*D*, calculated by fitting Eq. (S1) to the experimental data]. PFG‐NMR plots, that is, log‐attenuation plots as shown in Figures 6 a–c, provide a visual representation of the diffusion properties of the guest molecules within the porous media being studied. The lack of any evident curvature for PFG‐NMR plots, as well as the relatively large root‐mean‐square displacement (RMSD) values (Table S12),^23^ which are much greater than the average crystal size of the zeolite particles, indicates that the guest molecules have the time to explore the overall pore structure of the solid samples (both inter‐ and intra‐crystalline space) during the observation times used for the experiments (i.e., 200 ms for *n*‐octane/cumene and 500 ms for TIPB). Therefore, the calculated diffusion coefficients (*D*) represent the averaged molecular diffusivity across the whole zeolite particle.^24^ Consequently, the diffusion measured is not solely intracrystalline diffusion, but rather an effective diffusivity comprised of diffusion within the intracrystalline pore space and the pore space between crystallites, commonly referred to as long‐range diffusion. By fitting PFG‐NMR plots using Eq. (S1), the relevant diffusion coefficient of the systems under investigation was obtained (the negative value of the slope is equivalent to the numerical value *D* of the guest molecules being studied), as shown in Table S13. Interestingly, PFG‐NMR measurements showed that *D* values of the probing molecules in ZSM‐5‐P‐0.1‐6 were the smallest in comparison with the microporous AS‐MFI and C‐ZSM‐5. As a consequence, the pore network tortuosity, defined as the ratio of the bulk diffusivity of the guest molecule and that of the same molecule within the pore space, [*τ*, Figure 6 d, calculated using Eq. (S3)] of the ZSM‐5‐P‐0.1‐6 sample is the largest. Considering the kinetic diameter of *n*‐octane (0.43 nm) and cumene (0.68 nm), for ZSM‐5‐P‐0.1‐6, the comparatively small value of *D* and large value of *τ*, suggest that the developed method created a new percolating network within ZSM‐5‐P‐0.1‐6 zeolite crystals. As a result, the probing molecules gain access to the newly formed percolating network in the intra‐crystalline pores, which is more tortuous than the inter‐crystalline space, hence leading to lower values of the averaged measured diffusion coefficient due to increased collisions with the intra‐crystalline pore walls. Conversely, for the microporous AS‐MFI and C‐ZSM‐5, the probing molecules diffuse primarily within the inter‐crystalline pore space (since the intra‐crystalline space is less accessible due to the much smaller pore size of 0.54 nm). To prove this further, PFG‐NMR experiments were carried out using a bulky molecule, TIPB (kinetic diameter=0.94 nm),^25^ which is not able to enter the micropores of the intra‐crystalline space (i.e., the intrinsic micropores of ZSM‐5 zeolites). It was found that, when bulky TIPB was used as the probing species, (i) unlike *n*‐octane and cumene, PFG‐NMR plots of the materials are comparable for all the zeolite samples (Figure 6 c) and (ii) the rate of diffusion within the ZSM‐5‐P‐0.1‐6 sample measured across the whole zeolite particle increases, leading to a significant decrease in *τ* values, which becomes comparable with values measured for the parent and conventional zeolites (Table S13). Such finding can be clearly explained by considering the larger size of TIPB (in comparison with *n*‐octane and cumene), which hinders the access to the newly formed percolating network inside the crystalline space of ZSM‐5‐P‐0.1‐6 zeolites, hence the probe molecules experience a faster diffusion, lower tortuosity, within the inter‐crystalline space only. This is also confirmed by the comparable *D* and *τ* values of the zeolites under investigation when TIPB was used as the probing molecule in PFG‐NMR measurements (Table S13). In comparison with the state‐of‐the‐art post‐synthetic alkaline treatments (with or without SDA, Tables S14 and S15), the method produces ZSM‐5 nanoboxes with (i) a well‐developed percolating *meso*‐micro‐porous network and (ii) high concentration of strong acid sites (i.e., low SAR) in zeolite crystals.

## Conclusion

ZSM‐5 zeolites are important catalysts for many catalytic conversions such as catalytic cracking (to increase propylene selectivity by cracking gasoline range molecules selectively), MTO, alkylation, and ethanol dehydration. Accessibility issues and mass transfer limitations in ZSM‐5’s microporous framework commonly affect the outcome of the catalytic reaction to a great extent. Post‐synthetic desilication treatment of ZSM‐5 is the easiest way to introduce mesoporosity to ZSM‐5 zeolites but is limited by SAR of the parent zeolites. This work presents a simple and novel strategy by rapid ageing of the sol gel mixture to prepare the parent zeolite with tetrahedral EFAL and low SAR of about 12, which can be subsequently reconstructed (during the post‐synthetic treatment using SDA, that is, TPAOH) to give mesoporous hollow ZSM‐5 nanoboxes with low SAR of ≈16. The developed protocol removed the limitation of SAR of the parent zeolite on properties of the post‐treated ZSM‐5. The unique combination of the hierarchical *meso*‐micro‐porosity and low SAR of such mesoporous ZSM‐5 led to (i) the significantly improved accessibility of guest molecules to the active sites (evidenced by PFG‐NMR measurements) and (ii) comparably high yet stable catalytic performance in cracking reactions, which is important for the development of specific propylene‐selective catalysts aiming to improve the current on‐purpose propylene production technologies.

## Conflict of interest

The authors declare no conflict of interest.

## Supporting information

As a service to our authors and readers, this journal provides supporting information supplied by the authors. Such materials are peer reviewed and may be re‐organized for online delivery, but are not copy‐edited or typeset. Technical support issues arising from supporting information (other than missing files) should be addressed to the authors.

Supplementary
