# Supplementary material for: Creation of Al‐Enriched Mesoporous ZSM‐5 Nanoboxes with High Catalytic Activity: Converting Tetrahedral Extra‐Framework Al into Framework Sites by Post Treatment
Source: Angew Chem Int Ed Engl. 2020 Mar 26;59(44):19478–86. doi: 10.1002/anie.202002416 (PMC7687177; doi:10.1002/anie.202002416)
Supplement: Supplementary file 1 — Supplementary [file ANIE-59-19478-s001.pdf]

Angewandte Chemie
Eine Zeitschrift der Gesellschaft Deutscher Chemiker

Supporting Information

Creation of Al-Enriched Mesoporous ZSM-5 Nanoboxes with High Catalytic Activity: Converting Tetrahedral Extra-Framework Al into Framework Sites by Post Treatment

Yilai Jiao, Luke Forster, Shaojun Xu, Huanhao Chen, Jingfeng Han, Xuqing Liu, Yangtao Zhou, Jinmin Liu, Jinsong Zhang, Jihong Yu, Carmine D'Agostino,* and Xiaolei Fan**

anie_202002416_sm_miscellaneous_information.pdf

Supporting Information

Contents

Synthesis of Materials 2
Synthesis of mesoporous ZSM-5 nanoboxes 2
Post-synthetic treatment of AS-MFI using concentrated TPAOH solutions (0.3 and 0.5 M) 2
Synthesis and post-synthetic treatment of conventional ZSM-5 2
Synthesis of conventional hollow ZSM-5 with a SAR of ~45 (C-HO-ZSM-5) 3
Characterisation of Materials 3
Powder X-ray diffraction (XRD) 3
Nitrogen (N_2) adsorption-desorption 3
Solid-state nuclear magnetic resonance (NMR) 4
Scanning electron microscopy (SEM) and energy dispersive X-ray spectroscopy (EDX) 4
High resolution transmission electron microscopy (HRTEM) 4
Inductively coupled plasma-atomic emission spectroscopy (ICP-AES) 4
X-ray photoelectron spectroscopy (XPS) 4
Ammonia temperature-programmed desorption (NH_3-TPD) 4
Pyridine Fourier transform infrared (py-FTIR) 5
Thermogravimetric analysis (TGA) 5
Pulsed-Field Gradient Nuclear Magnetic Resonance (PFG-NMR) Diffusion Experiments 5
Catalysis, Stability and Regeneration 6
Cracking reactions 6
Hydrothermal steam aging of zeolite 7
Catalyst regeneration 7
Supporting Figures and Tables for Results and Discussion 7
References 21

Synthesis of Materials

Synthesis of mesoporous ZSM-5 nanoboxes

All chemicals were obtained from Sinopharm Chemical Reagent Co., Ltd and used as received. The procedure to prepare the parent zeolite is described as: aluminium chloride ($AlCl_3$) was added to a mixture of tetrapropylammonium hydroxide (TPAOH) and deionised (DI) water and continually stirred for 1 h at room temperature (RT), then the mixture was heated to 40 °C. Subsequently, tetraethylorthosilicate (TEOS) was added rapidly into the mixture under vigorous stirring and continuously stirred for 30 min until the synthesis gel become clear. The molar composition of the sol gel mixture is SiO_2:0.04Al_2O_3:0.3TPAOH:19H_2O. After rapid aging of the sol gel synthesis mixture, it was transferred into a Teflon-lined stainless-steel (SS) autoclave (500 ml capacity) for crystallisation at 160 °C (for different h). The resulting product was recovered by centrifugation and washed with DI water (100 mL for each wash, 3 times), then dried at 110 °C (overnight) and calcined in static air at 550 °C with for 6 h (heating rate = 1 °C min^{-1}). The as-synthesised parent zeolite is denoted as AS-MFI. For comparison, conventional ZSM-5 (denoted as C-ZSM-5) was also prepared using the conventional aging method (*i.e.* precursor aging at RT for 24 h), then followed by the same hydrothermal synthesis and work-up.

ZSM-5 nanoboxes are synthesised using a post-synthetic treatment method. Post-synthetic hydrothermal treatment of AS-MFI using TPAOH aqueous solution was carried by mixing the sample (4 g) with TPAOH solution (0.1 M, 40 mL, pH ≈ 13) at 400 rpm (for 20 min to form a slurry). Then the slurry was transferred to a Teflon-lined SS autoclave (100 ml) and left at autogenous pressure and 160 °C under hydrostatric condition for various h (specifically, 6, 12, 24, 48 and 96 h). Finally, the mixture was filtered, washed with DI water, dried at 120 °C for 12 h, and calcined at 550 °C for 6 h. The post-treated sample was denoted as ZSM-5-P-*x*-*y*, in which P represents *via* post-synthetic treatment, *x* represents the TPAOH concentration using in the post-treatment (in M) and *y* represents post-treatment time (in h), respectively.

Post-synthetic treatment of AS-MFI using concentrated TPAOH solutions (0.3 and 0.5 M)

Post-synthetic treatment of AS-MFI using 0.3 and 0.5 M TPAOH aqueous solutions was carried by mixing AS-MFI (4 g) with TPAOH solution (0.3 M and 0.5 M, 40 mL) at 400 rpm (for 20 min to form a slurry). Then the slurry was transferred to a Teflon-lined SS autoclave (100 ml) and left at autogenous pressure and 160 °C for 24 h. Finally, the mixture was filtered, washed with DI water, dried at 120 °C for 12 h, and calcined at 550 °C for 6 h. The treated sample was denoted as ZSM-5-P-0.3-24 and ZSM-5-P-0.5-24. Relevant pH values of the systems are ~14.

Synthesis and post-synthetic treatment of conventional ZSM-5

Standard procedure was used to synthesise the conventional ZSM-5 zeolite (denoted as C-ZSM-5), and it is described as: $AlCl_3$ was added to a mixture of TPAOH and DI water and continually stirred for 1 hour at RT. Subsequently, TEOS was added dropwise into the mixture under vigorous stirring at ice bath temperature (~12 h) and continuously stirred for 12 h at the same temperature. The molar composition of the sol gel mixture is SiO_2:0.04Al_2O_3:0.3TPAOH:19H_2O. After the 24 h aging of the sol gel synthesis mixture at RT, it was transferred into a Teflon-lined SS autoclave (500 ml capacity) for crystallisation at 160 °C for 48 h. The resulting product was recovered by centrifugation and washed with DI water (100 mL for each wash, 3 times, then dried at 110 °C (overnight) and calcined in static air at 550 °C (with a ramp rate of 1 °C min^{-1} for 6 h) to obtain C-ZSM-5.

The post-synthetic treatment of C-ZSM-5 using 0.1 M TPAOH aqueous solution was carried by mixing the C-ZSM-5 (4 g) with TPAOH solution (0.1 M, 40 mL) at 400 rpm (for 20 min to form a slurry). Then the slurry was transferred to a Teflon-lined SS autoclave (100 ml) and left at autogenous pressure and 160 °C for 6 h. Finally, the mixture was filtered, washed with DI water, dried at 120 °C for 12 h, and calcined at 550 °C for 6 h. The resulting sample of the post-treated C-ZSM-5 was denoted as C-ZSM-5-P-0.1-6 (6).

Synthesis of conventional hollow ZSM-5 with a SAR of ~45 (C-HO-ZSM-5)

The procedure to prepare the relevant parent ZSM-5 is described as: $AlCl_3$ was added to a mixture of TPAOH and DI water and continually stirred for 1 h at RT. Subsequently, TEOS was added into the mixture under vigorous stirring and continuously stirred for 24 h. The molar composition of the precursor sol is SiO_2:0.0125Al_2O_3:0.3TPAOH:19H_2O. After the 24 h aging of the precursor sol at RT, it was transferred into a Teflon-lined SS autoclave (500 ml capacity) for crystallisation at 160 °C for 48 h. The resulting product was recovered by centrifugation and washed with DI water (100 mL for each wash, 3 times, then dried at 110 °C (overnight) and calcined in static air at 550 °C with a ramp rate of 1 °C min^{-1} for 6 h.

The post-synthetic treatment of the parent zeolite using 0.7 M TPAOH aqueous solution was carried by mixing the sample (4 g) with TPAOH aqueous solution (0.7 M, 40 mL) at 400 rpm (for 20 min to form a slurry). Then the slurry was transferred to a Teflon-lined SS autoclave (100 ml) and left at autogenous pressure and 160 °C for 24 h. Finally, the mixture was filtered, washed with DI water, dried at 120 °C for 12 h, and calcined at 550 °C for 6 h. The treated sample was denoted as C-HO-ZSM-5.

Characterisation of Materials

Powder X-ray diffraction (XRD)

XRD patterns of materials were recorded using a PANalytical X'Pert Pro system fitted with $CuK\alpha_1$ X-ray source (λ = 0.15406 nm) operated at 40 kV and 40 mA. The measurement was performed over a 2θ range of 5°–35° in 0.02 step size at a scanning rate of 1° min^{-1}. The relative crystallinity (RC) of zeolites was determined by using a standard Integrated Peak Area Method, which involves a comparison of the integrated peak areas in the range of 22.5 to 25.0° 2θ. Details of the method has been described elsewhere.[1] The sample with the strongest peak at 23.1° 2θ was selected as the reference, which was ZSM-5-P-0.1-12 in this study. It is worth mentioning that, in this work, the parent zeolite, *i.e.* AS-MFI, was not perfectly crystallised with the non-coordinated tetrahedral Al and Si species, and hence the crystallinity of AS-MFI is comparatively poor. The subsequent post-treatment with the aqueous TPAOH solution (at 0.1 M) under hydrothermal conditions can facilitate the re-crystallisation of the parent AS-MFI zeolite during the post-treatment, and hence producing the relevant ZSM-5-P zeolites with the improved crystallinity.

Nitrogen (N_2) adsorption-desorption

N_2 physisorption measurements at the liquid nitrogen temperature (−196.15 °C) using a Micromeritics 3Flex surface area and pore size analyser. Prior to the measurement, the sample (~100 mg) was degassed at 350 °C under vacuum overnight. The micropore size distribution was

calculated by the Horvath-Kawazoe (HK) method, and mesopore size distribution was determined from the adsorption branch of the isotherms by the Barrett-Joyner-Halenda (BJH) method.

Solid-state nuclear magnetic resonance (NMR)

Solid-state NMR spectra were recorded on an Agilent 600 DD2 spectrometer operating at a Larmor frequency of 600 MHz for 1H. ^{29}Si magic angle spinning (MAS) NMR spectra were recorded at 119.15 MHz (with the proton decoupling (TPPM) during acquisition) using a 3.6 μs pulse with a 30 s recycle delay and 1024 scans (tetramethylsilane as the reference). ^{27}Al MAS NMR spectra were recorded at 156.25 MHz (with the proton decoupling (TPPM) during acquisition) using a 3.6 μs pulse with a 5 s recycle delay and 512 scans (aluminium chloride as the reference).

Scanning electron microscopy (SEM) and energy dispersive X-ray spectroscopy (EDX)

SEM-EDX analysis was performed on a JEOL 7401 high-resolution field emission scanning electron microscope with an Oxford INCA 350 EDX system.

High resolution transmission electron microscopy (HRTEM)

HRTEM analysis of the microstructure of zeolites was performed using a Fei Tecnai F20 field emission gun transmission electron microscope at 200 kV. Before TEM analysis, the sample was dispersed in ethanol (which was sonicated for 5 min in an ultrasonic bath), and a droplet of the solution was casted on a carbon-coated mesh grid.

Inductively coupled plasma-atomic emission spectroscopy (ICP-AES)

ICP-AES analysis of various zeolites for the elemental analysis of their Al and Si contents was performed on a Varian Vista AX ICP-AES spectrometer. To prepare the solutions for ICP-AES analysis, the zeolite sample (0.1 g) was dissolved using hydrofluoric acid (HF) solution (10 wt.% HF in water, 5 ml) at RT, then diluted using DI water to 100 ml.

X-ray photoelectron spectroscopy (XPS)

XPS analysis was carried out using ThermoScientific ESCALAB 250 spectrometer equipped with monochromated AlKα X-ray source (1486.6 eV, 150W, spot size = 500 μm), a charge neutraliser and a hemispherical electron energy analyser. During data acquisition, the chamber pressure was kept below 10^{-9} mbar. The spectra were analysed using the CasaXPS software pack and corrected for charging using C1s binding energy (BE) as the reference at 284.8 eV.

Ammonia temperature-programmed desorption (NH_3-TPD)

NH_3-TPD measurements were performed using a Micromeritics AutoChem II 2920 chemisorption analyser (Micromeritics, USA) to determine the acidic property of the zeolites. ~100 mg zeolite was pre-treated at 823 K for 1 h and then cooled down to 323 K under Helium (He). A gas mixture of NH_3 in He (10%:90%, 30 cm^3 min^{-1}) was then introduced to saturate the catalyst followed by the purge of pure He (60 cm^3 min^{-1}) at 373 K for 2 h to remove the physically adsorbed NH_3. Finally, NH_3-TPD was performed by heating the catalyst from 373 K to 873 K with a heating rate of 10 K min^{-1} under He flow (30 cm^3 min^{-1}) and the desorbed NH_3 was monitored by a gas chromatography (GC) equipped with a thermal conductivity detector (TCD).

Pyridine Fourier transform infrared (py-FTIR)

py-FTIR analysis was performed using a nexus Model Infrared Spectrophotometer (Termo Nicolet Co, USA) operating at 2 cm^{-1} full width at half maximum (FWHM) equipped with an *in situ* cell containing CaF_2 windows. Adsorption of pyridine was performed at RT and then evacuated at 200 °C measuring pyridine adsorbed at all acid sites. After that, the sample was evacuated *in situ* at 350 °C corresponding to the pyridine adsorption at the strong acid sites.

Thermogravimetric analysis (TGA)

TGA of the used zeolites from the cracking reaction was performed using a TG analyser (Q600 TGA-DSC, TA Instruments, Germany) under air (flow rate = 100 ml min^{-1}). The temperature ramp was from RT to 800 °C with the heating rate of 10 °C min^{-1}.

Pulsed-Field Gradient Nuclear Magnetic Resonance (PFG-NMR) Diffusion Experiments

Zeolites were pelletised with particle sizes of 1.6–1.8 mm for PFG-NMR measurements. The zeolite particles were dried at 60 °C in a vacuum oven overnight and calcined at 550 °C for 12 h. To prepare samples for PFG-NMR experiments, the zeolite particles were soaked in *n*-octane, cumene or 1,3,5-triisopropylbenzene (TIPB) for 2 days to ensure full saturation of the porous matrix of the zeolite samples with the respective probe molecule. The saturated samples were then dried on a filter paper to remove any excess liquid from the external surface of the particles and transferred to 5 mm NMR tubes. To minimise relevant errors due to evaporation of the volatile liquid, a small amount of pure liquid was dropped onto a filter paper, which was placed under the cap of the NMR tube. The tube was then placed into the magnet and left for approximately 15 min to achieve thermal equilibrium before measurements started. NMR experiments were performed on a Magritek SpinSolve benchtop NMR spectrometer operating at a ^1H frequency of 43 MHz. PFG-NMR experiments were carried out using a diffusion probe capable of producing magnetic field gradient pulses up to 163 mT m^{-1}. Diffusion measurements were performed using the pulsed-field gradient stimulated echo sequence (PGSTE sequence).[2] The sequence is made by combining a series of radiofrequency pulses (RF) with magnetic field gradients (*g*, Figure S1). The NMR signal attenuation of PFG-NMR experiments as a function of the gradient strength, $E(g)$, is related to the experimental variables and the diffusion coefficient (D) by:

$$\frac{E(g)}{E_0} = \exp\left[-D \cdot \gamma_H^2 \cdot g^2 \cdot \delta^2 \cdot \left(\Delta - \frac{\delta}{3}\right)\right] \quad \text{(S1)}$$

where E_0 is the NMR signal in the absence of gradient, γ_H is the gyromagnetic ratio of the nuclei being studied (i.e. ^1H in this case), g is the strength of the gradient pulse of duration δ, and Δ is the observation time (i.e. the time interval between the leading edges of the gradient pulses). In Eq. S1, it is often convenient to define the product $\gamma_H^2 \cdot g^2 \cdot \delta^2 \cdot \left(\Delta - \frac{\delta}{3}\right)$ as the *b*-factor, hence the Equation can be written as $\frac{E(g)}{E_0} = \exp[-D \cdot b]$.

The measurements using cumene and octane were performed by fixing Δ = 200 ms and δ = 3 ms, and measurements with TIPB were performed by fixing Δ = 500 ms and δ = 5 ms. The magnitude of g was varied linearly with 16 spaced increments. To achieve full signal attenuation, maximum values of g of up to 163 mT m^{-1} were necessary. All the measurements were performed at atmospheric pressure and 25 °C. The diffusion coefficients D were calculated by fitting Eq. S1 to the experimental data.

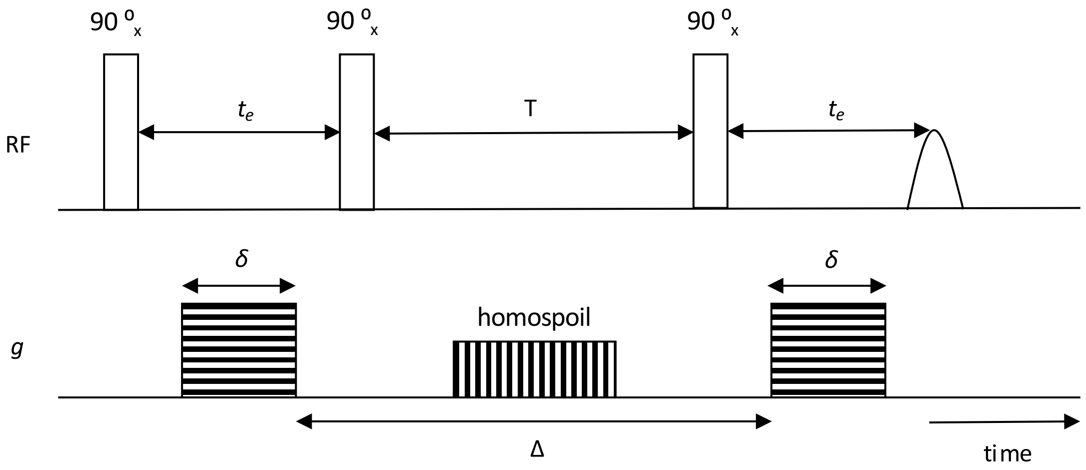

Figure S1. PGSTE pulse sequence showing gradient pulse duration δ, echo time t_e, storage interval T, homospoil gradient and diffusion time, Δ.

Root-mean-square displacement (RMSD) values of the probing molecules are determined using Eq. S2.

$$\mathrm{RMSD} = \sqrt{2 \times D \times t_e} \tag{S2}$$

To understand the diffusion process occurring within the pore structures under investigation, the PFG interaction parameter is calculated,[3] which in the case of using hydrocarbons as probe species, can be approximated to the tortuosity of the pore network, τ, defined as in Eq. S3.[4]

$$\tau = \frac{D}{D_{bulk}} \tag{S3}$$

where D is the diffusivity of probing molecules within the zeolite samples, and D_{bulk} is the diffusivity of the bulk liquid.

Catalysis, Stability and Regeneration

Cracking reactions

Cracking of *n*-octane and cumene over the catalyst was carried out in a fixed bed reactor (I.D. = 10mm). The packed bed consists of 1 g pelletised zeolite catalysts (particle size = 1.6–1.8 mm) with glass beads as dilution. For catalytic cracking, the catalyst bed was preheated at the reaction

temperature for 2 h, then *n*-octane (0.1 ml min^{-1} with N_2 as the carrier gas at 240 ml min^{-1}) or cumene (0.1 ml min^{-1} with N_2 as the carrier gas at 150 ml min^{-1}) was introduced to start the reaction at the same temperature (*i.e.* 540 °C for *n*-octane and 320 °C for cumene). The gaseous products of the reactions were analysed using an in-line gas chromatograph (GC, Agilent 7890B) equipped with a flame ionisation detector (FID) and Agilent PoraPLOT Q column. The carbon balance is above 95%.

Hydrothermal steam aging of zeolite

Hydrothermal aging of ZSM-5 nanoboxes (~100 mg) using steam was carried out in a tube furnace (Carbolite Gero Ltd., UK) at 500 °C. N_2 (at 200 mL min^{-1}) was used as the carrier gas which was passed through a bubbler containing DI water (at 50 °C) to generate steam. The sample were treated by steaming for 10 h prior to XRD and N_2 physisoprtion analysis.

Catalyst regeneration

Regeneration of the used ZSM-5-P-0.1-6 zeolite catalyst (the used catalyst is denoted as ZSM-5-P-0.1-6-U, the ZSM-5-P-0.1-6-U catalyst was recovered from of *n*-octane cracking) was performed by calcining ZSM-5-P-0.1-6-U at 550 °C in O_2 (10 vol.%) in N_2 (at 200 mL min^{-1}) for 6 h. The regenerated ZSM-5-P-0.1-6-U was assessed in catalytic cracking of *n*-octane and characterised by N_2 physisorption, NH_3-TPD and MAS NMR.

Supporting Figures and Tables for Results and Discussion

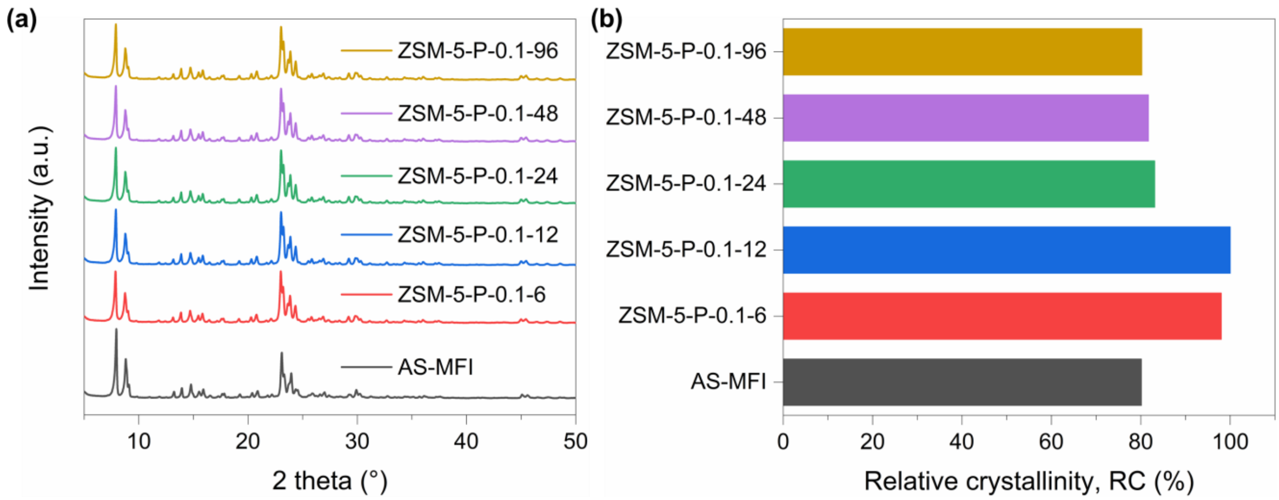

Figure S2. (a) XRD patterns and (b) relative crystallinity (RC) of AS-MFI and mesoporous ZSM-5 nanoboxes (*i.e.* ZSM-5-P zeolites) from the post-synthetic TPAOH (0.1 M) treatment of AS-MFI with different treatment times (6–96 h, ZSM-5-P-0.1-12 as the reference with 100% RC).

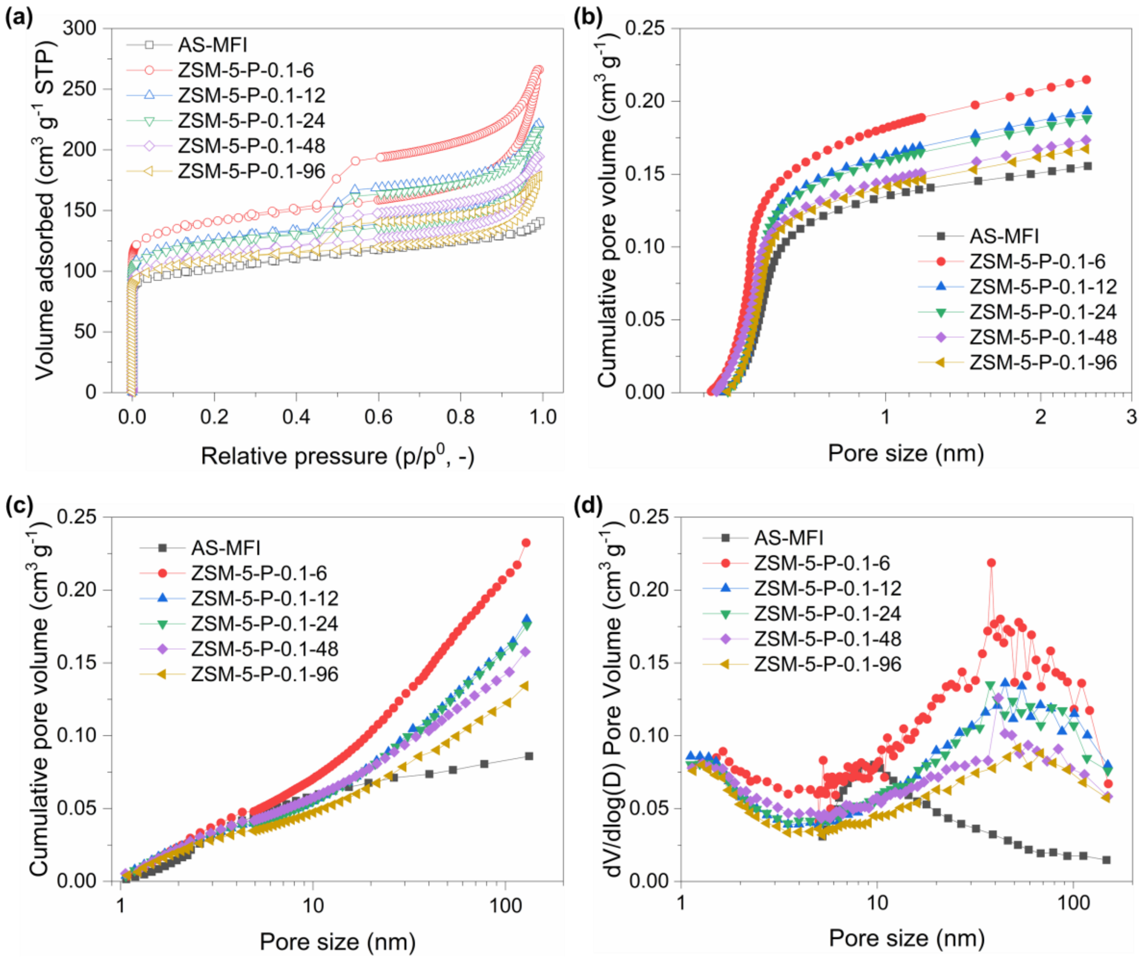

Figure S3. (a) Nitrogen (N_2) adsorption/desorption isotherms, (b) microprore cumulative pore volume, (c) mesopore cumulative pore volume and (d) differential pore size distribution of AS-MFI and mesoporous ZSM-5 nanoboxes (*i.e.* ZSM-5-P zeolites) from the post-synthetic TPAOH (0.1 M) treatment of AS-MFI with different treatment times (6–96 h).

Table S1. Textural properties and molar SARs of AS-MFI and mesoporous ZSM-5 nanoboxes (*i.e.* ZSM-5-P zeolites) from the post-synthetic TPAOH (0.1 M) treatment of AS-MFI with different treatment times (6–96 h).

Sample	S_{BET} [$m^2\,g^{-1}$]	S_{micro} [$m^2\,g^{-1}$]	$S_{ext.}$ [$m^2\,g^{-1}$]	V_{micro} [$cm^3\,g^{-1}$]	V_{total} [$cm^3\,g^{-1}$]	V_{meso} [$cm^3\,g^{-1}$]	Molar SAR [-]		
							by XPS	by EDX	by ICP
AS-MFI	375	275	100	0.12	0.22	0.10	5.9	12.1	12.3
ZSM-5-P-0.1-6	521	372	149	0.15	0.41	0.26	10.9	16.3	15.6
ZSM-5-P-0.1-12	468	339	129	0.14	0.34	0.20	9.1	16.6	16.3
ZSM-5-P-0.1-24	456	331	125	0.14	0.34	0.20	10.2	17.6	17.1
ZSM-5-P-0.1-48	420	295	125	0.12	0.30	0.18	12.5	15.2	16.4
ZSM-5-P-0.1-96	406	293	113	0.12	0.28	0.16	12.6	14.7	15.2

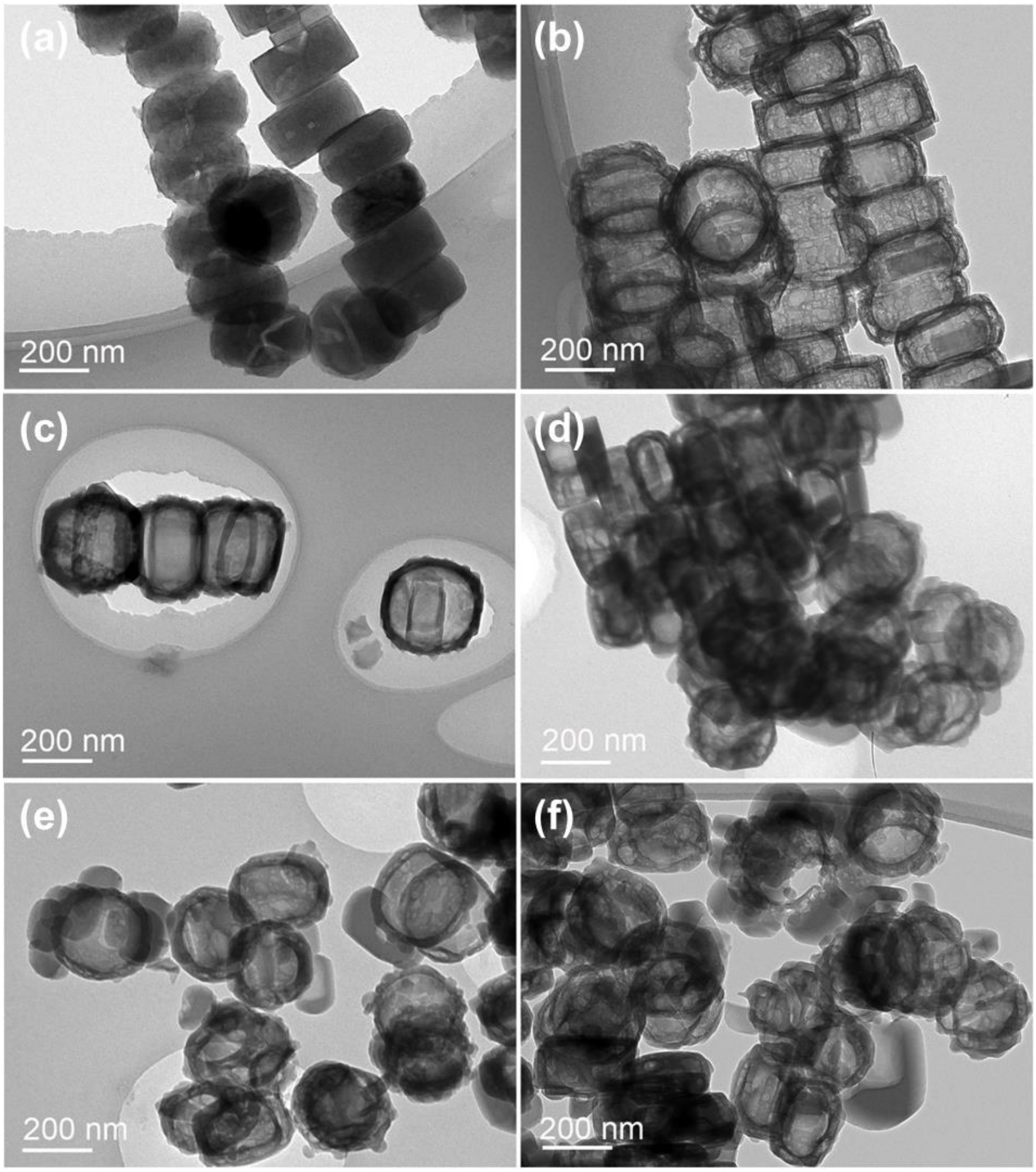

Figure S4. TEM micrographs of (a) AS-MFI, (b) ZSM-5-P-0.1-6, (c) ZSM-5-P-0.1-12, (d) ZSM-5-P-0.1-24, (e) ZSM-5-P-0.1-48 and (f) ZSM-5-P-0.1-96 zeolites.

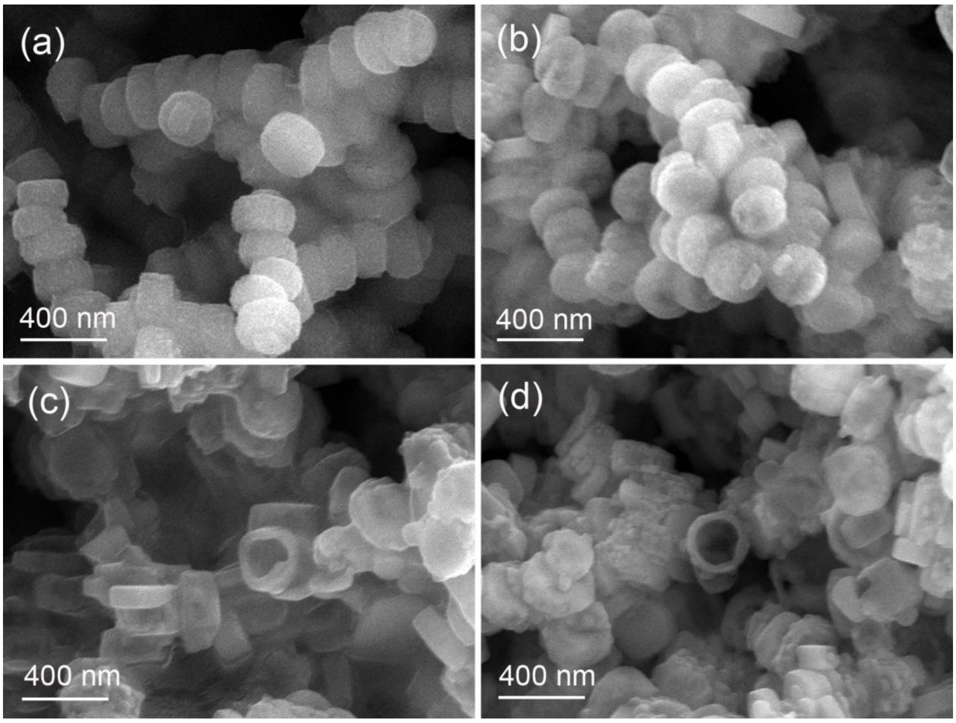

Figure S5. SEM micrographs of (a) ZSM-5-P-0.1-12, (b) ZSM-5-P-0.1-24, (c) ZSM-5-P-0.1-48 and (d) ZSM-5-P-0.1-96 zeolites.

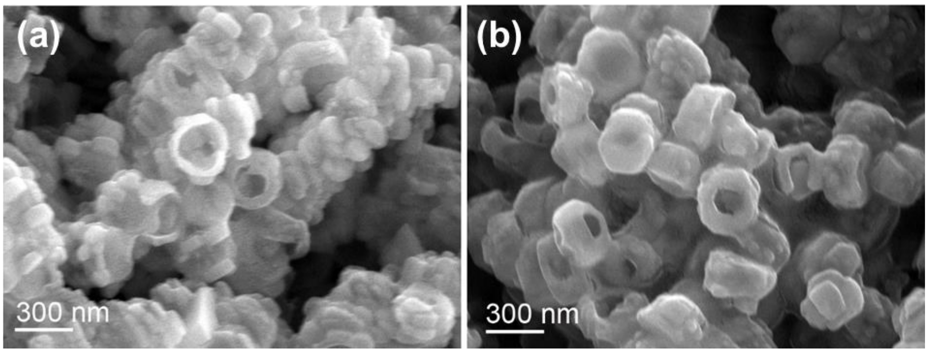

Figure S6. SEM micrographs of (a) ZSM-5-P-0.3-24 and (b) ZSM-5-P -0.5-24 zeolites (the samples were obtained by treating AS-MFI using 0.3 M and 0.5 M TPAOH solutions for 24 h).

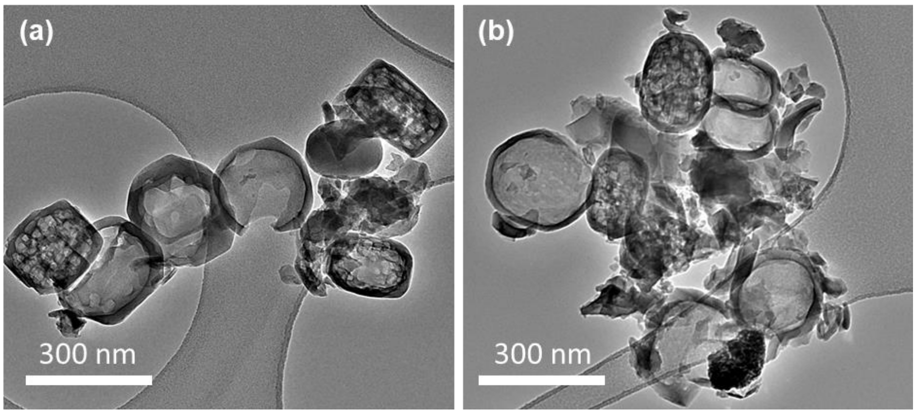

Figure S7. TEM micrographs of (a) ZSM-5-0.3-P-24 and (b) ZSM-5-P-0.5-24 zeolites.

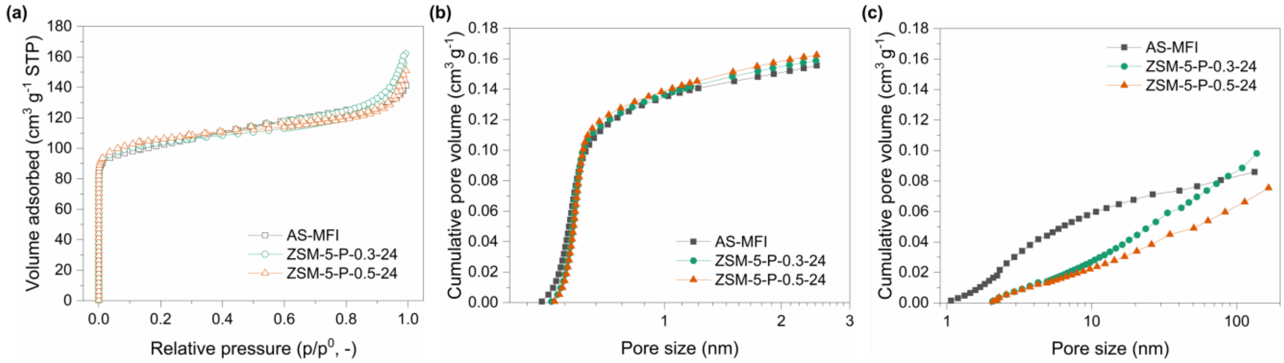

Figure S8. (a) N_2 adsorption/desorption isotherms, (b) microprore cumulative pore volume, (c) mesopore cumulative pore volume of AS-MFI, ZSM-5-P-0.3-24 and ZSM-5-P-0.5-24 zeolites.

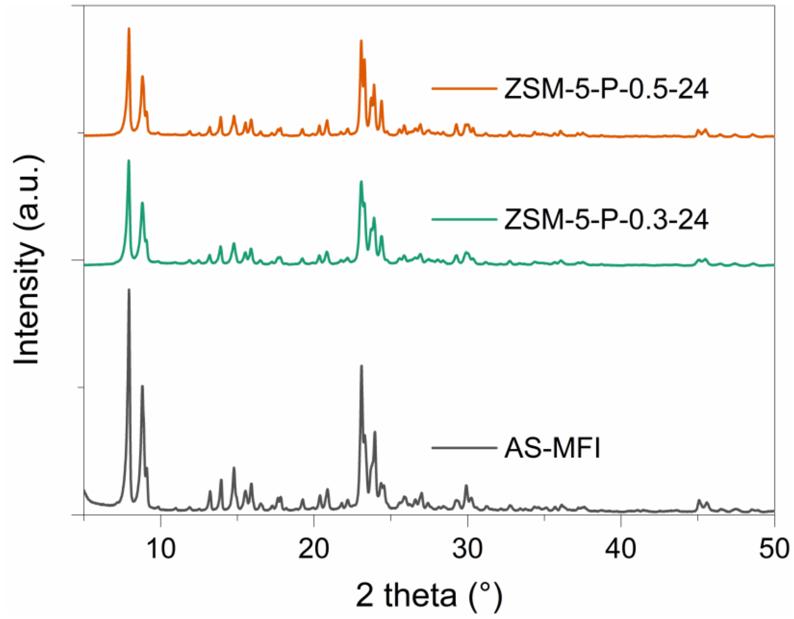

Figure S9. XRD patterns of AS-MFI, ZSM-5-P-0.3-24 and ZSM-5-P-0.5-24 zeolites.

Table S2. Textural properties, molar SARs and relative crystallinities (RC) of AS-MFI, P-ZSM-5-0.3-24 and P-ZSM-5-0.5-24 zeolites.

Sample	S_{BET}	S_{micro}	$S_{ext.}$	V_{micro}	V_{total}	Molar SAR [-]			RC
	[$m^2\ g^{-1}$]	[$m^2\ g^{-1}$]	[$m^2\ g^{-1}$]	[$cm^3\ g^{-1}$]	[$cm^3\ g^{-1}$]	by XPS	by EDX	by ICP	[%]
AS-MFI	375	275	100	0.12	0.22	5.9	12.1	12.3	80.1
ZSM-5-P-0.3-24	392	317	75	0.13	0.25	15.4	15.6	14.9	55.9
ZSM-5-P-0.5-24	388	302	86	0.13	0.23	13.9	13.7	14.3	51.0
C-ZSM-5	341	285	56	0.14	0.21	9.7	13	13.4	-
C-ZSM-5-0.1-6	337	261	76	0.12	0.23	12.0	16.5	16.1	77.3
C-HO-ZSM-5	379	234	145	0.10	0.28	42.0	45.1	44.9	-

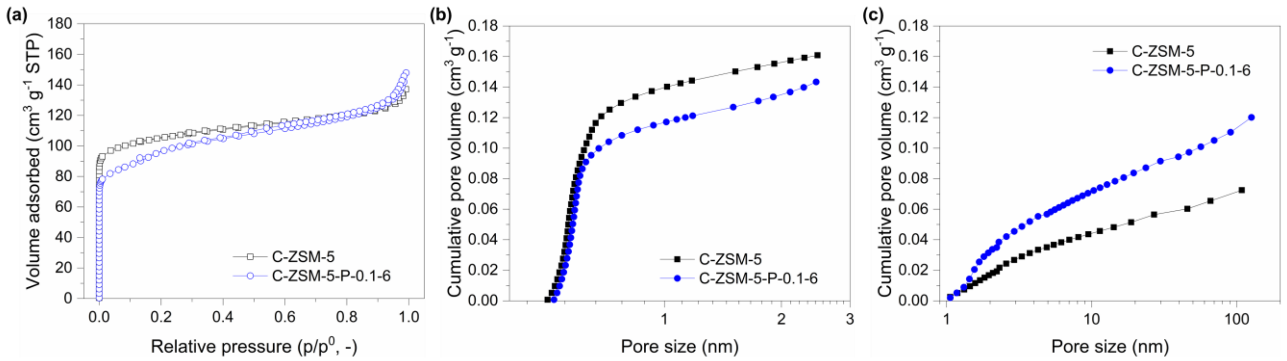

Figure S10. (a) N_2 adsorption/desorption isotherms, (b) microprore cumulative pore volume, (c) mesopore cumulative pore volume of C-ZSM-5 and C-ZSM-5-P-0.1-6 zeolites (C-ZSM-5-P-0.1-6 was obtained from the post-synthetic TPAOH (0.1 M) treatment of C-ZSM-5 for 6 h).

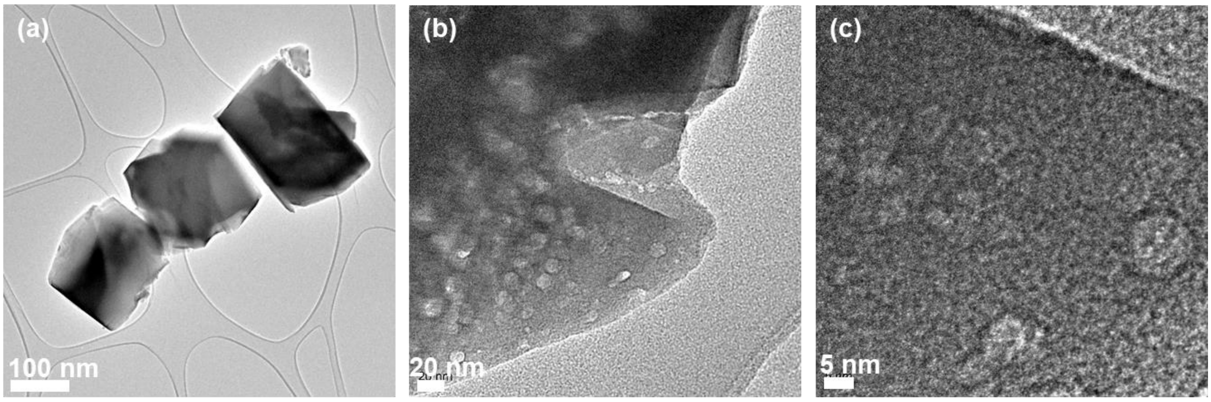

Figure S11. TEM micrographs of (a) C-ZSM-5 and (b–c) C-ZSM-5-P-0.1-6 zeolites.

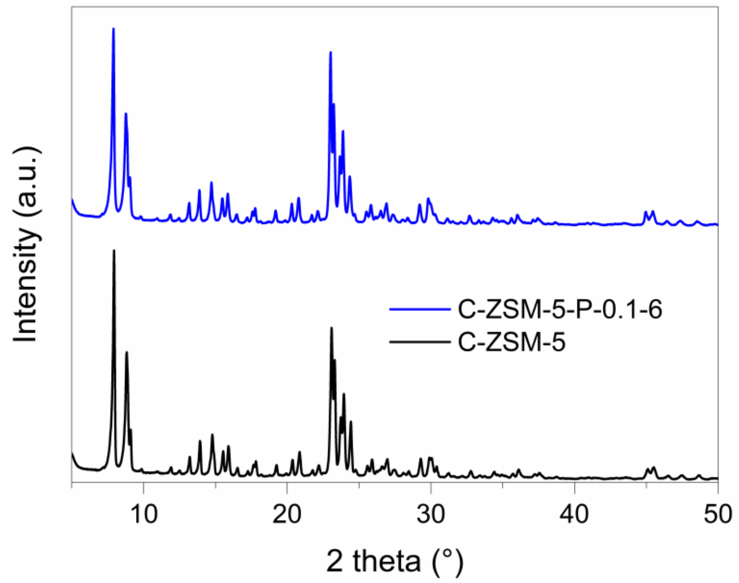

Figure S12. XRD patterns of C-ZSM-5 and C-ZSM-5-P-0.1-6 zeolites.

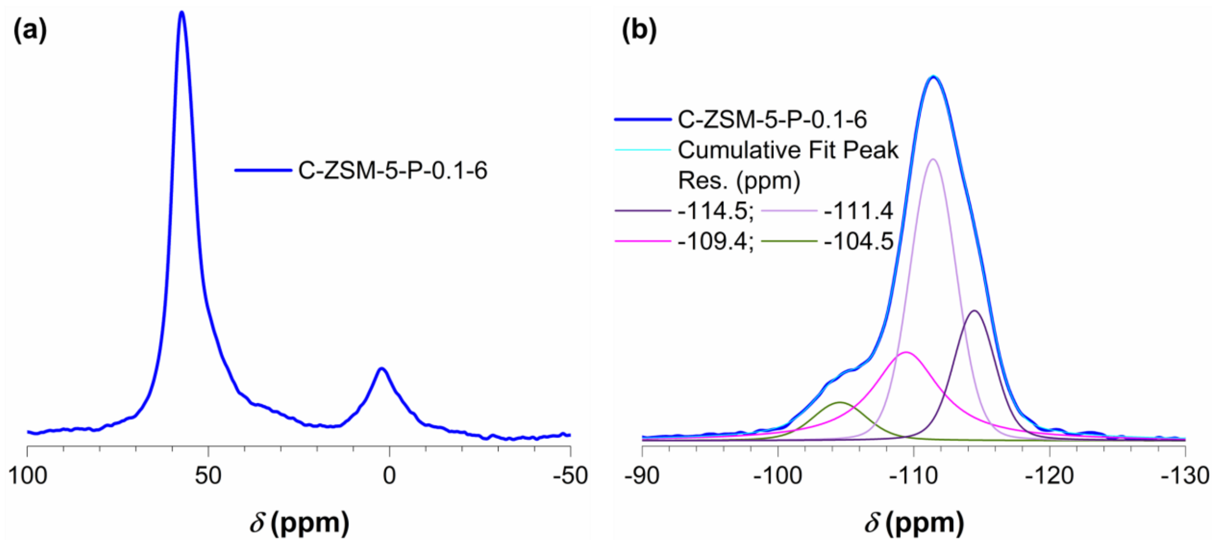

Figure S13. (a) ^{27}Al MAS NMR spectrum and (b) ^{29}Si MAS NMR spectra of the C-ZSM-5-P-0.1-6 zeolite.

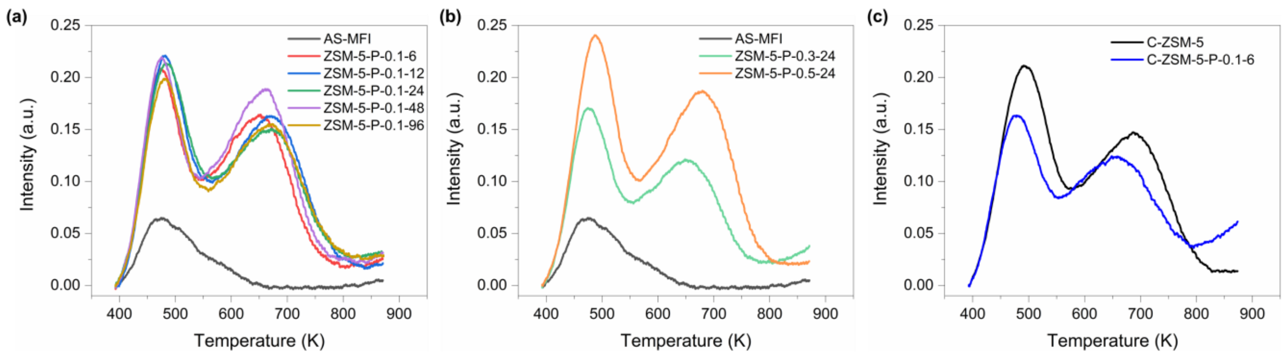

Figure S14. Comparative NH_3-TPD curves of (a) AS-MFI and mesoporous ZSM-5 nanoboxes (*i.e.* ZSM-5-P zeolites), (b) AS-MFI, ZSM-5-P-0.3-24 and ZSM-5-P-0.5-24 zeolites, and (c) C-ZSM-5 and C-ZSM-5-P-0.1-6 zeolites.

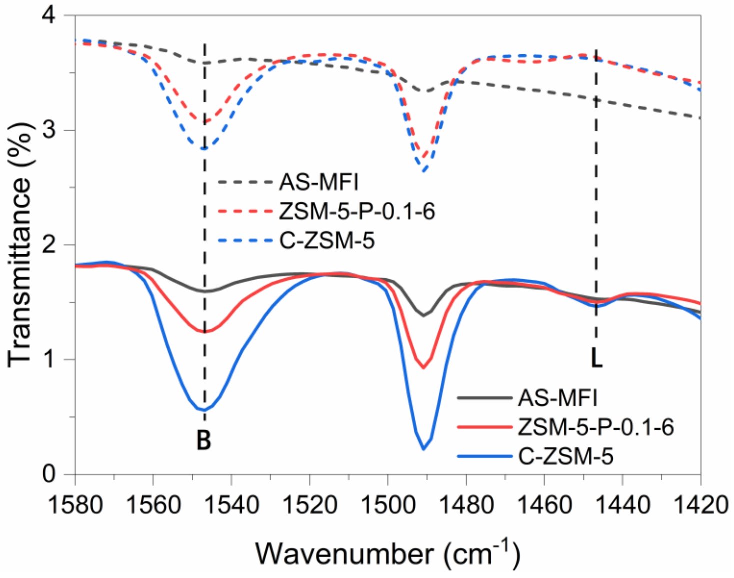

Figure S15. IR spectra, in the region characteristic of adsorbed pyridine vibrations, of the relevant zeolite catalysts after pyridine sorption and evacuation at 200 °C (solid lines) and 350 °C (dash lines).

Table S3. Acidic properties of the zeolite catalysts under investigation by NH_3-TPD.

Sample	Temperature at maximum [K]		Weak acidity [μmol g^{-1}]	Strong acidity [μmol g^{-1}]
	First peak	Second peak		
AS-MFI	473	569	107.6	24.1
ZSM-5-P-0.1-6	471	645	249.6	403.1
ZSM-5-P-0.1-12	478	666	292.9	425.4
ZSM-5-P-0.1-24	480	662	293.3	364.7
ZSM-5-P-0.1-48	472	652	248.7	448.7
ZSM-5-P-0.1-96	477	661	248.2	382.6
ZSM-5-P-0.3-24	474	648	208.9	286.6
ZSM-5-P-0.5-24	485	675	303.1	469.2
C-ZSM-5	488	682	321.6	393.6
C-ZSM-5-P-0.1-6	474	651	215.1	282.3
C-HO-ZSM-5	468	640	131.1	214.4

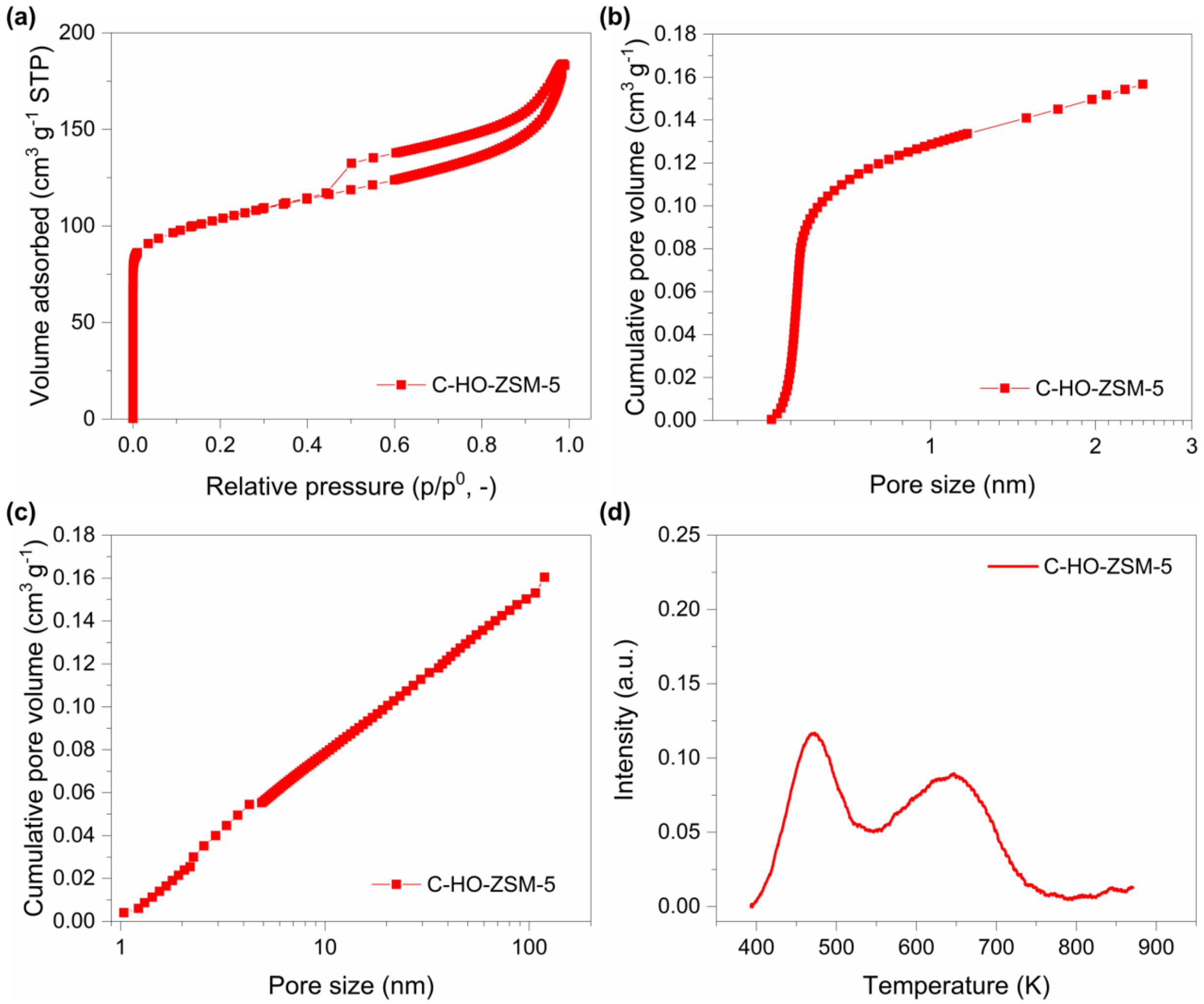

Figure S16. (a) N_2 adsorption-desorption (open symbols) isotherms, (b) microprore cumulative pore volume, (c) mesopore cumulative pore volume, and (d) NH_3-TPD curves of C-HO-ZSM-5 (SAR = ~45).

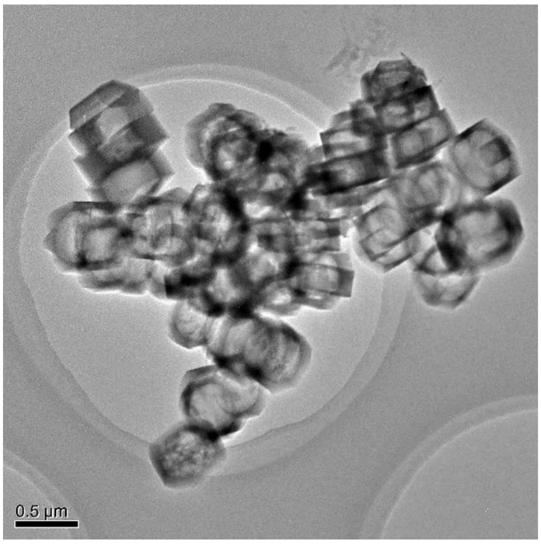

Figure S17. TEM micrographs of C-HO-ZSM-5 (SAR = ~45).

Table S4. Key Physical and chemical properties of the zeolite catalysts used for cracking reactions.

Catalysts	S_{BET} [m^2 g^{-1}]	S_{micro} [m^2 g^{-1}]	$S_{ext.}$ [m^2 g^{-1}]	V_{micro} [cm^3 g^{-1}]	V_{total} [cm^3 g^{-1}]	SAR[a] [-]	Acidity[b] [µmol g^{-1}]
AS-MFI	375	275	100	0.12	0.22	12.3	24.1
ZSM-5-P-0.1-6	521	372	149	0.15	0.41	15.6	403.1
C-ZSM-5	341	285	56	0.14	0.21	13.4	393.6
C-HO-ZSM-5	379	234	145	0.10	0.28	44.9	214.4

[a] Molar SAR values by ICP; [b] Strong acidity by NH_3-TPD.

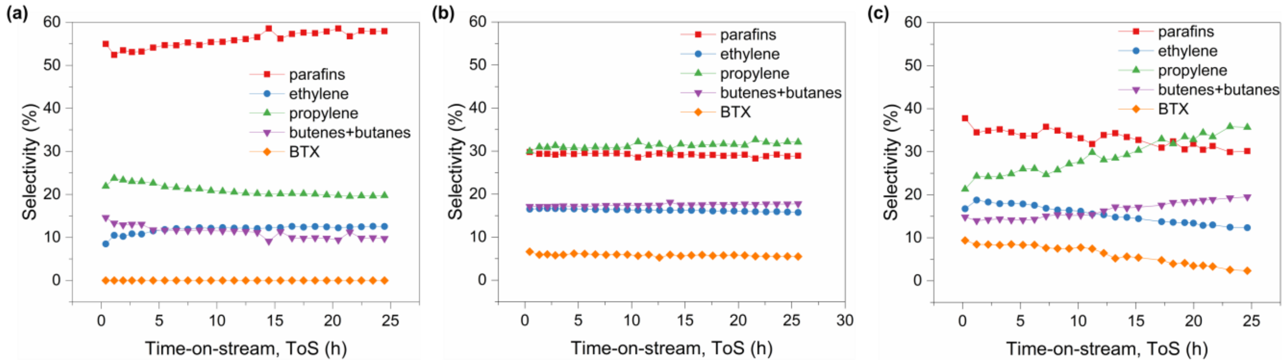

Figure S18. Selectivity of *n*-octane cracking as a function of time-on-stream over (a) AS-MFI, (b) ZSM-5-P-0.1-6 and (c) C-ZSM-5 zeolites.

Table S5. Conversions and selectivity of *n*-octane cracking reactions (at 540 °C) over the zeolite catalysts under study.[a]

Zeolite	Conversion[b] [%]	Selectivity [%]					P/E[c] [-]
		parafins	ethylene	propylene	butenes+butanes	BTX	
AS-MFI	3.2	53.9	10.2	22.7	13.3	0.00	2.2
ZSM-5-P-0.1-6	72.7	29.3	16.6	30.7	17.2	6.2	1.8
ZSM-5-P-0.1-6 (regenerated)	72.8	29.8	16.8	30.0	17.3	6.1	1.8
ZSM-5-P-0.1-24	75.2	30.7	16.9	27.1	16.2	9.0	1.6
ZSM-5-P-0.1-48	82.3	31.8	17.6	25.9	16.2	8.5	1.5
ZSM-5-P-0.1-96	88.5	33.4	17.7	22.3	14.2	12.3	1.3
C-ZSM-5	90.0	35.8	17.9	23.2	14.3	8.8	1.3
C-HO-ZSM-5	31.0	29.3	9.0	38.1	23.5	0.0	4.2

[a] All data are the averaged values of the first three hours on stream with the margin of errors of *ca.* 7% for C-ZSM-5 and <1.5% for the rest; [b] carbon mass balances >95.0%; [c] the propylene/ethylene (P/E) ratio.

Table S6. Conversion and selectivity of cumene cracking reactions (at 320 °C) over the zeolite catalysts under study.[a]

Catalyst	Conversion[a] [%]	Selectivity [%]				
		parafins	ethylene	propylene	butenes+butanes	BTX
ZSM-5-P-0.1-6	94.0	7.0	0.7	7.3	11.3	73.8
C-ZSM-5	66.1	6.06	0.7	7.7	10.1	75.3
C-HO-ZSM-5	83.4	5.0	0.4	11.7	11.1	71.8

[a] All data are the averaged values of the first three hours on stream with the margin of errors of *ca.* 21% for C-ZSM-5, *ca.* 5% for C-HO-ZSM-5 and 0.3% for ZSM-5-P-0.1-6; [b] carbon mass balances >95.0%.

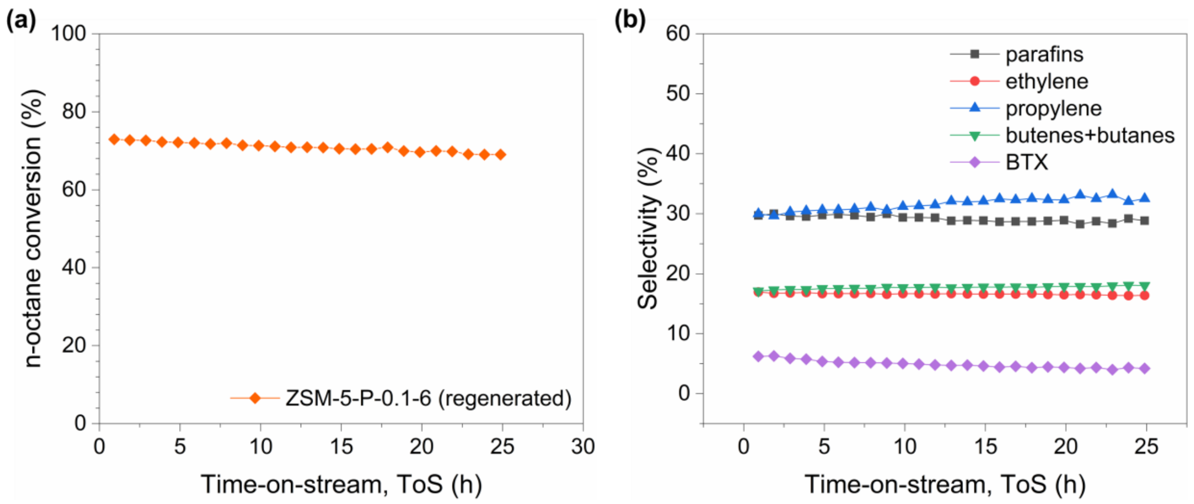

Figure S19. (a) conversion and (b) selectivity of *n*-octane cracking over the regenerated ZSM-5-P-0.1-6-U zeolite catalyst.

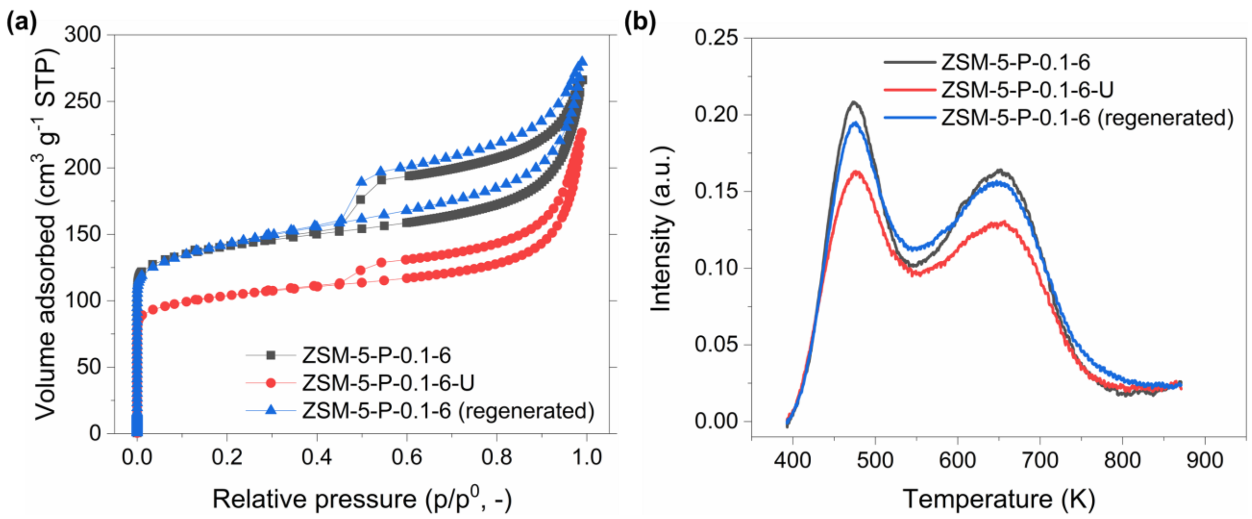

Figure S20. (a) N_2 adsorption/desorption isotherms and (b) NH_3-TPD curves of the ZSM-5-P-0.1-6, ZSM-5-P-0.1-6-U and regenerated ZSM-5-P-0.1-6 zeolite catalysts.

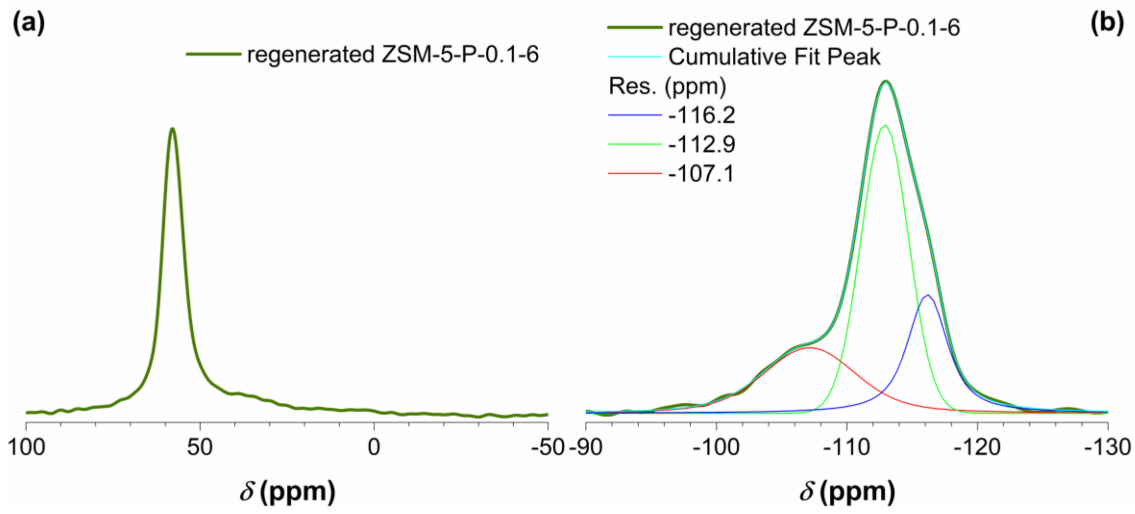

Figure S21. (a) ^{27}Al MAS NMR and (b) ^{29}Si MAS NMR spectra of the regenerated ZSM-5-P-0.1-6 zeolite.

Table S7. Comparative textural properties of the fresh, used and regenerated ZSM-5-P-0.1-6 zeolite catalyst by N_2 physisorption.

Sample	S_{BET} [m^2 g^{-1}]	S_{micro} [m^2 g^{-1}]	$S_{ext.}$ [m^2 g^{-1}]	V_{micro} [cm^3 g^{-1}]	V_{total} [cm^3 g^{-1}]
ZSM-5-P-0.1-6	521	372	149	0.15	0.41
ZSM-5-P-0.1-6-U	382	265	117	0.11	0.35
Regenerated ZSM-5-P-0.1-6	522	369	153	0.15	0.43

Table S8. Comparative acidic properties of the fresh, used and regenerated ZSM-5-P-0.1-6 zeolite catalyst by NH_3-TPD.

Sample	Temperature at maximum [K]		Weak acidity [μmol g^{-1}]	Strong acidity [μmol g^{-1}]
	First peak	Second peak		
ZSM-5-P-0.1-6	471	645	249.6	403.1
ZSM-5-P-0.1-6-U	471	645	221.7	313.2
Regenerated ZSM-5-P-0.1-6	471	642	250.6	397.1

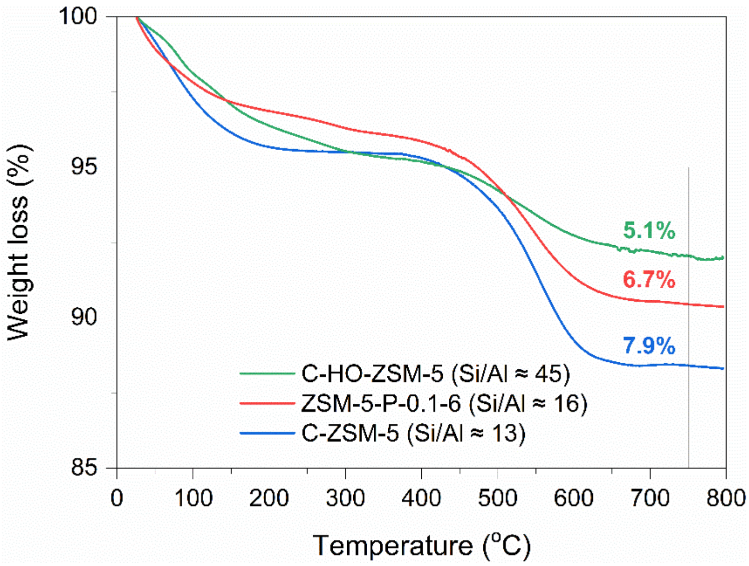

Figure S22. Weight loss of the used zeolite catalysts (by TGA) from the cumene cracking reactions.

Table S9. Comparative textural properties of the fresh and used ZSM-5-P-0.1-6 (SAR = ~16), C-HO-ZSM-5 (SAR = ~45) and C-ZSM-5 (SAR = ~13) from the catalytic cracking reactions by N_2 physisorption.

Sample	S_{BET} [$m^2\ g^{-1}$]	S_{micro} [$m^2\ g^{-1}$]	$S_{ext.}$ [$m^2\ g^{-1}$]	V_{micro} [$cm^3\ g^{-1}$]	V_{total} [$cm^3\ g^{-1}$]
ZSM-5-P-0.1-6	521	372	149	0.15	0.41
ZSM-5-P-0.1-6-U[a]	382	265	117	0.11	0.35
ZSM-5-P-0.1-6-U[b]	249	188	61	0.08	0.31
C-HO-ZSM-5	379	234	145	0.10	0.28
C-HO-ZSM-5-U[a]	304	193	111	0.10	0.20
C-HO-ZSM-5-U[b]	213	178	35	0.08	0.20
C-ZSM-5	341	285	56	0.14	0.21
C-ZSM-5-U[a]	92	75	17	0.03	0.05
C-ZSM-5-U[b]	20	16	4	0.01	0.02

[a] The used zeolite from *n*-octane cracking; [b] the used zeolite from cumene cracking.

Table S10. Comparative acidic properties of the fresh and used zeolite catalysts (from cracking reactions) by NH_3-TPD.

Sample	Temperature at maximum [K]		Weak acidity[a] [μmol g^{-1}]	Strong acidity[b] [μmol g^{-1}]
	First peak	Second peak		
ZSM-5-P-0.1-6	471	645	249.6	403.1
ZSM-5-P-0.1-6U[a]	471	645	221.7	313.2
ZSM-5-P-0.1-6U[b]	475	640	192.8	228.3
C-HO-ZSM-5	468	640	131.1	214.4
C-HO-ZSM-5-U[a]	478	666	102.9	168.4
C-HO-ZSM-5-U[b]	465	605	74.5	100.6
C-ZSM-5	488	682	321.6	393.6
C-ZSM-5-U[a]	472	637	146.6	172.0
C-ZSM-5-U[b]	480	669	117.6	104.9

[a] The used zeolite from *n*-octane cracking; [b] the used zeolite from cumene cracking.

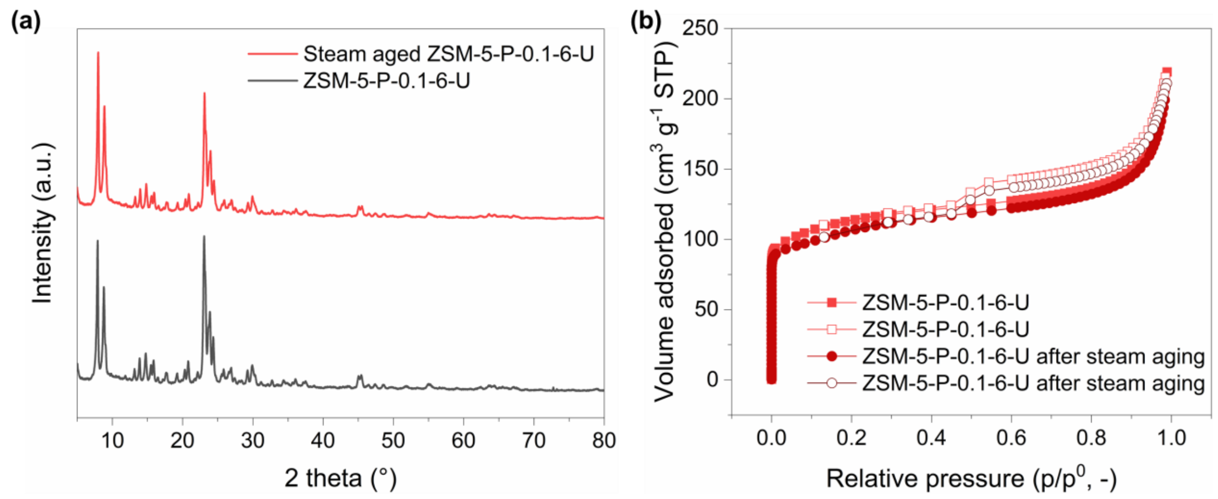

Figure S23. (a) XRD patterns and (b) N_2 adsorption/desorption isotherms of ZSM-5-P-0.1-6-U before and after the steam aging.

Table S11. Textural properties ZSM-5-P-0.1-6-U (from the catalytic *n*-octane cracking) and its counterpart after steam aging treatment.

Sample	S_{BET} [m^2 g^{-1}]	S_{micro} [m^2 g^{-1}]	$S_{ext.}$ [m^2 g^{-1}]	V_{micro} [cm^3 g^{-1}]	V_{total} [cm^3 g^{-1}]
ZSM-5-P-0.1-6-U	415	262	153	0.11	0.34
Steam aged ZSM-5-P-0.1-6-U	391	244	147	0.10	0.33

Table S12. Root mean squared displacements (RMSD) of *n*-octane, cumene and 1,3,5-triisopropylbenzene (TIPB) imbibed within the ZSM-5-P-0.1-6, AS-MFI and C-ZSM-5 zeolite catalysts.

Sample	$RMSD_{OCT}$ [μm]	$RMSD_{CUM}$ [μm]	$RMSD_{TIPB}$ [μm]
C-ZSM-5	23.0	18.6	11.4
AS-MFI	18.9	17.7	11.1
ZSM-5-P-0.1-6	12.1	11.6	8.2

Table S13. Self-diffusivity and diffusivity values of bulk *n*-octane, cumene and TIPB, and *n*-octane and cumene imbibed within the C-ZSM-5, AS-MFI and ZSM-5-P-0.1-6 samples, and the calculated tortuosity values of the samples.

Sample	D_{OCT} [m^2 s^{-1}] × 10^{9}	τ_{OCT}	D_{CUM} [m^2 s^{-1}] × 10^{9}	τ_{CUM}	D_{TIPB} [m^2 s^{-1}] × 10^{9}	τ_{TIPB}
C-ZSM-5	1.29±1 %	1.82±1 %	0.80±1 %	1.95±1 %	0.13±1 %	2.00±1 %
AS-MFI	0.76±8 %	2.68±8 %	0.70±1 %	2.22±1 %	0.14±1 %	1.86±1 %
ZSM-5-P-0.1-6	0.30±5 %	6.62±5 %	0.22±8 %	7.33±8 %	0.12±2 %	2.17±2 %
Bulk liquid	2.30±1 %	-	1.56±1 %	-	0.07±4 %	3.71±4 %

Table S14. Information of the parent MFI zeolites and the resulting zeolites prepared using the conventional post-synthetic alkaline treatment methods.

Parent zeolites			Post-treatment conditions	Resulting mesoporous zeolites		Ref.
zeolite	SAR	Crystal size		Textural property	SAR	
ZSM-5	40	μm aggregates	0.2 M NaOH at 80 °C (5 h)	Mesoporous	n/a	[5]
ZSM-5	37	n.a.	0.05 M NaOH at 50 or 70 °C (0.5–30 h)	Mesoporous	374–2133	[6]
ZSM-5	37	μm aggregates	0.2 M NaOH at 65 °C (0.5 h)	Mesoporous	24	[7]
ZSM-5	37	μm aggregates	0.2 M NaOH at 35–85 °C (15–120 min)	Mesoporous	24–37	[8]
ZSM-5	17	n.a.	0.2 M NaOH at 65 °C (30 min)	Limited mesoporosity	15	[9]
	19			Limited mesoporosity	18	
	26			Mesoporous	18	
	37			Mesoporous	24	
	42			Mesoporous	29	
	176			Mesoporous	133	
	1038			Limited mesoporosity	560	
ZSM-5	26	400–700 nm	0.2 M NaOH at 65 °C (15 or 30 min)	Mesoporous and hollow	18–19	[10]
	41	20–30 μm		Mesoporous and hollow	32	
ZSM-5	72	200–300 nm	0.6 M Na_2CO_3 at 80°C for 36 h.	Hollow structure	49	[11]
ZSM-5	50	<100 nm	0.1 M NaOH at 80°C (2–5 min, 1–10 h)	Hollow structure	27–41	[12]
	14	μm aggregates	0.1 M NaOH at 80°C for one week	Limited mesoporosity	n/a	

Table S15. Information of the parent MFI zeolites and the resulting zeolites prepared using the conventional post-synthetic methods using TPAOH solutions.

Parent zeolites			Post-treatment conditions	Resulting mesoporous zeolites		Ref.
zeolite	SAR	Crystal size		Textural property	SAR	
silicalite-1	-	<200 nm	0.56 M TPAOH with additional Al sources ($Al(NO_3)_3$) at 170 °C (0.75–24 h)	Hollow structure	50–100	[13]
silicalite-1	-	0.5–1 μm	0.56 M TPAOH with $Al(NO_3)_3$ at 170 °C (24 h)	Heterogeneous mesoporosity	125	
silicalite-1	-	μm		Mesoporous	33–402	[14]
ZSM-5	100	<300 nm	1 M TPAOH at 170 °C (24 h)	Hollow structure	101–105	[15]
ZSM-5	40–79	<200 nm	TPAOH (0.05–0.5 M) at 170 °C (72 h)	Hollow structure	47–70	[16]
ZSM-5	50	300–500 nm	TPAOH (0.15 or 0.3 M) at 170 °C (72 h)	Hollow structure	n/a	[17]
ZSM-5	37	n.a.	TPAOH (0.3 M) at 170 °C (72 h)	Hollow structure	33	[18]
silicalite-1	-	200–900 nm	0.3 M TPAOH with $NaAlO_2$ at 170 °C (72 h) with different liquid-to-solid ratios	Hollow structure	41–63	[19]
silicalite-1	-	<100 nm	TPAOH with $NaAlO_2$ at 170 °C (72 h) with different synthesis times	Hollow structure	31–51	[20]

References

[1] X. Ou, S. Xu, J. M. Warnett, S. M. Holmes, A. Zaheer, A. A. Garforth, M. A. Williams, Y. Jiao, X. Fan, *Chem. Eng. J.* **2017**, *312*, 1–9.

[2] E. O. Stejskal, J. E. Tanner, *J. Chem. Phys.* **1965**, *42*, 288–292.

[3] C. D'Agostino, J. Mitchell, L. F. Gladden, M. D. Mantle, *J. Phys. Chem. C* **2012**, *116*, 8975–8982.

[4] E. L. Perkins, J. P. Lowe, K. J. Edler, N. Tanko, S. P. Rigby, *Chem. Eng. Sci.* **2008**, *63*, 1929–1940.

[5] O. Masaru, S. Shin-ya, T. Junko, N. Yasuto, K. Eiichi, M. Masahiko, *Chem. Lett.* **2000**, *29*, 882–883.

[6] T. Suzuki, T. Okuhara, *Microporous Mesoporous Mater.* **2001**, *43*, 83–89.

[7] J. C. Groen, L. A. A. Peffer, J. A. Moulijn, R. Pérez, x, J. rez, *Microporous Mesoporous Mater.* **2004**, *69*, 29–34.

[8] J. C. Groen, L. A. A. Peffer, J. A. Moulijn, J. Pérez-Ramírez, *Colloids Surfaces A* **2004**, *241*, 53–58.

[9] J. C. Groen, J. C. Jansen, J. A. Moulijn, J. Perez-Ramirez, *J. Phys. Chem. B* **2004**, *108*, 13062–13065.

[10] J. C. Groen, T. Bach, U. Ziese, A. M. Paulaime-van Donk, K. P. de Jong, J. A. Moulijn, J. Pérez-Ramírez, *J. Am. Chem. Soc.* **2005**, *127*, 10792–10793.

[11] C. Mei, Z. Liu, P. Wen, Z. Xie, W. Hua, Z. Gao, *J. Mater. Chem.* **2008**, *18*, 3496–3500.

[12] D. Fodor, F. Krumeich, R. Hauert, J. A. van Bokhoven, *Chem. Eur. J.* **2015**, *21*, 6272–6277.

[13] Y. Wang, A. Tuel, *Microporous Mesoporous Mater.* **2008**, *113*, 286–295.

[14] D. Verboekend, J. Pérez-Ramírez, *Chem. Eur. J.* **2011**, *17*, 1137–1147.

[15] S. Li, A. Tuel, D. Laprune, F. Meunier, D. Farrusseng, *Chem. Mater.* **2015**, *27*, 276–282.

[16] C. Dai, A. Zhang, M. Liu, X. Guo, C. Song, *Adv. Func. Mater.* **2015**, *25*, 7479–7487.

[17] Z. Hong, Z. Wang, D. Chen, Q. Sun, X. Li, *Appl. Surf. Sci.* **2018**, *440*, 1037–1046.

[18] J. Li, M. Liu, X. Guo, S. Zeng, S. Xu, Y. Wei, Z. Liu, C. Song, *Ind. Eng. Chem. Res.* **2018**, *57*, 15375–15384.

[19] Z. Ma, T. Fu, Y. Wang, J. Shao, Q. Ma, C. Zhang, L. Cui, Z. Li, *Ind. Eng. Chem. Res.* **2019**, *58*, 2146–2158.

[20] J. Shao, T. Fu, Z. Ma, C. Zhang, H. Li, L. Cui, Z. Li, *Catal. Sci. Technol.* **2019**, *9*, 6647–6658.
